# Expression of 16 Nitrogenase Proteins within the Plant Mitochondrial Matrix

**DOI:** 10.3389/fpls.2017.00287

**Published:** 2017-03-03

**Authors:** Robert S. Allen, Kimberley Tilbrook, Andrew C. Warden, Peter C. Campbell, Vivien Rolland, Surinder P. Singh, Craig C. Wood

**Affiliations:** ^1^CSIRO Agriculture and FoodCanberra, ACT, Australia; ^2^CSIRO Land and WaterCanberra, ACT, Australia

**Keywords:** nitrogenase, synthetic biology, nitrogen fixation, metabolic engineering, mitochondrial targeting

## Abstract

The industrial production and use of nitrogenous fertilizer involves significant environmental and economic costs. Strategies to reduce fertilizer dependency are required to address the world's increasing demand for sustainable food, fibers, and biofuels. Biological nitrogen fixation, a process unique to diazatrophic bacteria, is catalyzed by the nitrogenase complex, and reconstituting this function in plant cells is an ambitious biotechnological strategy to reduce fertilizer use. Here we establish that the full array of biosynthetic and catalytic nitrogenase (Nif) proteins from the diazotroph *Klebsiella pneumoniae* can be individually expressed as mitochondrial targeting peptide (MTP)-Nif fusions in *Nicotiana benthamiana*. We show that these are correctly targeted to the plant mitochondrial matrix, a subcellular location with biochemical and genetic characteristics potentially supportive of nitrogenase function. Although Nif proteins B, D, E, F, H, J, K, M, N, Q, S, U, V, X, Y, and Z were all detectable by Western blot analysis, the NifD catalytic component was the least abundant. To address this problem, a translational fusion between NifD and NifK was designed based on the crystal structure of the nitrogenase MoFe protein heterodimer. This fusion protein enabled equimolar NifD:NifK stoichiometry and improved NifD expression levels in plants. Finally, four MTP-Nif fusion proteins (B, S, H, Y) were successfully co-expressed, demonstrating that multiple components of nitrogenase can be targeted to plant mitochondria. These results establish the feasibility of reconstituting the complete componentry for nitrogenase in plant cells, within an intracellular environment that could support the conversion of nitrogen gas into ammonia.

## Introduction

Diazotrophic bacteria produce ammonia from N_2_ gas via biological nitrogen fixation (BNF), catalyzed by nitrogenase. Yet the demands of modern agriculture far outstrip this source of fixed nitrogen, and industrially-produced nitrogenous fertilizer (INF, Smil, 2002) is used extensively in agriculture. However, both INF production and application are causes of pollution (Good and Beatty, 2011) and considered overall unsustainable (Rockstrom et al., 2009). Approximately half the fertilizer applied worldwide is not taken up by crops (Cui et al., 2013; Kronzucker and Coskun, 2015), leading to fertilizer runoff, promotion of weeds and eutrophication of waterways (Good and Beatty, 2011). Resultant algal blooms reduce oxygen levels, causing environmental damage locally and offshore throughout coral reefs (Sutton et al., 2008; De'ath et al., 2012; Glibert et al., 2014). Furthermore, although over-fertilization is a problem in many developed countries, in certain regions fertilizer availability limits crop yields (Mueller et al., 2012).

Strategies to reduce the global dependence on nitrogen fertilizers need to be explored, and biotechnological approaches have been suggested. To this end, the notion of engineering plants capable of BNF has long attracted considerable interest (Merrick and Dixon, 1984), and has been the focus of recent reviews (Oldroyd and Dixon, 2014; de Bruijn, 2015). Potential approaches include (i) extending the symbiotic relationship of diazotrophs from legumes to cereals (Santi et al., 2013), (ii) re-engineering endosymbiotic microorganisms to be capable of nitrogen fixation (Geddes et al., 2015), and (iii) genetic engineering of nitrogenase into plant cells (Curatti and Rubio, 2014). Whilst all of these approaches are ambitious we outline here our first steps toward the direct engineering of nitrogenase into the mitochondrial matrix of plants.

Nitrogenase, the unique enzyme complex capable of BNF in diazotrophic bacteria, requires a multigene assembly pathway for its biosynthesis and function, reviewed extensively previously (Rubio and Ludden, 2008; Seefeldt et al., 2009; Hu and Ribbe, 2013). There are two subunits of the canonical iron-molybdenum nitrogenase, the catalytic MoFe subunit comprised of NifD and NifK proteins, and the electron donor Fe subunit comprised of NifH. A further series of proteins is involved in electron transport and nitrogenase assembly (maturation, scaffolding, co-factor insertion), including Nif B, M, S, U, E, N, X, V, J, Y, F, Z, and Q. Specific biochemical conditions are also required for nitrogenase assembly and function. Foremost among these is the necessity for protection from oxygen, as nitrogenase is extremely oxygen sensitive (Robson and Postgate, 1980). Furthermore, large amounts of ATP, reductant, readily available Fe, Mo, S-adenosylmethionine, and homocitrate are required for biosynthesis and function of the metalloprotein complex (Rubio and Ludden, 2008; Hu and Ribbe, 2013).

Given these requirements for nitrogenase function, the mitochondrial matrix is considered a suitable location for its reconstitution and activity (Curatti and Rubio, 2014). Significantly, the matrix possess oxygen-consuming enzymes that allow oxygen-sensitive enzymes to function, therefore this environment may be similarly permissive for nitrogenase activity. Secondly as the major site of plant metalloenzyme synthesis, mitochondria contain biosynthetic assembly proteins, which could provide the functionality of equivalent Nif proteins (Lill and Mühlenhoff, 2008; Balk and Pilon, 2011). These concepts have been partially validated by the successful isolation of an *ex vivo* active Nif Fe subunit from the mitochondrial matrix of aerobically grown yeast (López-Torrejón et al., 2016). This breakthrough result indicates that the matrix may support the assembly and activity of a complete nitrogenase. Nevertheless, an active nitrogenase in any eukaryote will require a much larger array of Nif proteins to be co-expressed, including the catalytic core.

As a first step toward reconstitution of nitrogenase in plant mitochondria, evidence is needed that individual Nif proteins can be correctly targeted to the organelle. For this purpose we made use of the model plant *Nicotiana benthamiana*, where metabolic engineers have developed an expression platform that allows single and multiple transgenes to be expressed and assayed within a week (Wood et al., 2009). As most matrix-located proteins are nuclear-encoded, we have relied upon recent advances in understanding of subcellular signaling and transport of proteins (Huang et al., 2009; Murcha et al., 2014), using a previously characterized N-terminal peptide targeting signal (Lee et al., 2012). Here we re-engineer 16 Nif proteins from diazotrophic *Klebsiella pneumoniae* for targeting to the plant matrix and assess their expression and processing in *N. benthamiana*. Our results are discussed in the context of recent advances in nitrogenase engineering in subcellular locations of plants and yeast.

## Results

### Validation of a mitochondrial targeting peptide for directing Nif proteins into *N. benthamiana* leaf mitochondria

**Figure 1:**
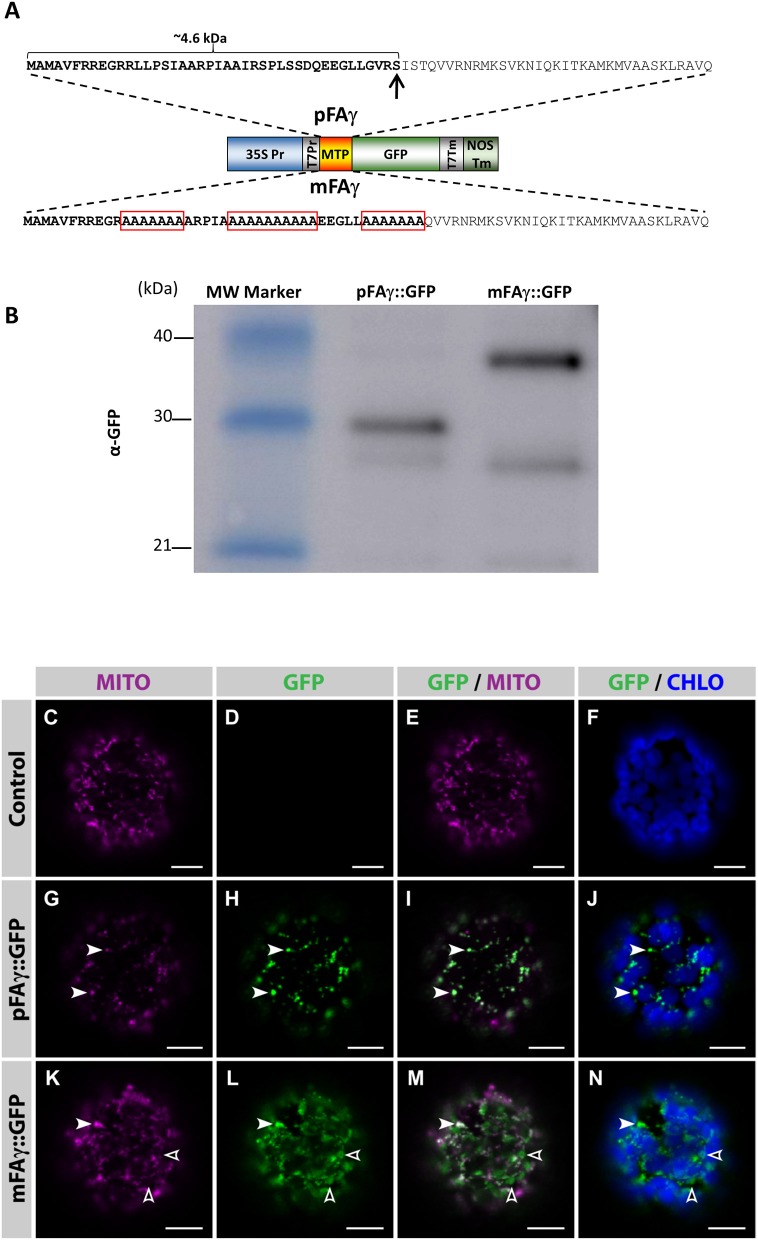
**Validation of the MTP for mitochondrial matrix targeting in ***N. benthamiana*** leaves**. **(A)** Schematic diagram of the constructs used to transiently express pFAγ::GFP fusion polypeptides in *N. benthamiana* leaves. The wild-type pFAγ mitochondrial targeting peptide sequence (MTP) shown above and the mutated version (mFAγ) shown below. Red boxes indicate regions of alanine substitutions. The arrow indicates the predicted point of cleavage by the MPP. 35S Pr, CaMV 35S promoter; T7Pr, T7 RNA polymerase promoter; MTP, pFAγ, or mFAγ region; GFP, GFP polypeptide; T7Tm, T7 RNA polymerase transcription terminator; NOSTm, 3′ transcription terminator/polyadenylation region of the *nos* gene. **(B)** Western blot of protein extracts for constructs expressing pFAγ::GFP or mFAγ::GFP fusion polypeptides in *N. benthamiana* leaves. Molecular weights of the markers in the first lane are indicated. The band in the pFAγ::GFP lane is the cleaved fusion polypeptide, whereas the intense band in the mFAγ::GFP lane is the uncleaved fusion polypeptide. The GFP antibody also produces a second slightly fainter lower band of ~28 kDa. **(C–N)** Laser scanning confocal microscopy images of protoplasts isolated 3 dpi from *N. benthamiana* leaves that were either non-infiltrated controls **(C–F)**, or infiltrated with pFAγ::GFP **(G–J)** or mFAγ::GFP **(K–N)**. In protoplasts expressing pFAγ::GFP **(G–J)**, the GFP signal fully co-localized with MitoTracker (white arrowheads), while in protoplasts expressing mFAγ::GFP **(K–N)**, the GFP signal only partially co-localized with MitoTracker (white arrowheads,) and a large fraction of GFP was mis-targeted to other subcellular areas (empty arrowheads). MITO, MitoTracker fluorescence; GFP, GFP fluorescence; GFP/MITO, overlay of GFP and MitoTracker staining fluorescence; GFP/CHLO overlay of GFP and chloroplast fluorescence. Scale bars: 10 μm.

An *Arabidopsis thaliana* F1-ATPase pFAγ subunit mitochondrial targeting peptide (MTP; pFAγ) that has previously been functionally validated in *Arabidopsis* protoplasts (Lee et al., 2012) was tested for its ability to traffic transgenic proteins to the matrix of intact plant leaf cells using the *N. benthamiana* transient leaf assay system. The entire pFAγ (77 AA) as previously described was translationally fused to the N terminus of GFP (pFAγ::GFP), where the first 42 AA (~4.6 kDa) of pre-sequence is predicted to be removed via the matrix-located peptidase (Figure 1A). As a control to discriminate matrix-processed GFP, several alanine amino acid substitutions were introduced in regions of the MTP required for its mitochondrial recognition and processing by the mitochondrial processing peptidase (MPP; Lee et al., 2012), thus this version (mFAγ::GFP) should be unable to be correctly processed and rather produce a full length fusion protein ~4.6 kDa larger than pFAγ::GFP. Five-week-old *N. benthamiana* leaves were infiltrated with either pFAγ::GFP or mFAγ::GFP. SDS-polyacrylamide gel electrophoresis (PAGE) and Western blots were carried out on crude protein extracted 4 days post infiltration (4 dpi) using a GFP antibody. For pFAγ::GFP, a band was observed corresponding to the expected size (~30 kDa) for matrix-processed GFP polypeptide, whereas for mFAγ::GFP, a higher MW band was observed (~35 kDa) of the expected size for unprocessed GFP fusion polypeptide (Figure 1B). A fainter band at ~28 kDa was observed for both pFAγ::GFP and mFAγ::GFP. This was not observed in negative controls and therefore may represent a degradation product, or possibility a product arising from alternative transcription or translation. To determine the subcellular localization of the GFP fusion proteins, protoplasts were isolated 3 dpi from leaf tissues containing either pFAγ::GFP, mFAγ::GFP, or no vector, and were examined by confocal microscopy after staining their mitochondria with MitoTracker. In protoplasts expressing pFAγ::GFP, the GFP signal fully co-localized with the mitochondrial marker (Figures 1G–J, white arrowheads) and was absent in control protoplasts (Figures 1C–F). In contrast, in protoplasts expressing mFAγ::GFP the GFP signal only partially localized to mitochondria (Figures 1K–N, white arrowheads), and a large fraction of the GFP signal was found to be targeted to other subcellular areas (Figures 1K–N, open arrowheads). Taken together these analyses indicate that the pFAγ MTP was capable of translocating the GFP fusion polypeptide to the matrix in *N. benthamiana* leaf cells and cleavage by the MPP, while the use of mFAγ MTP resulted in largely mis-targeted fusion proteins.

**Figure 2:**
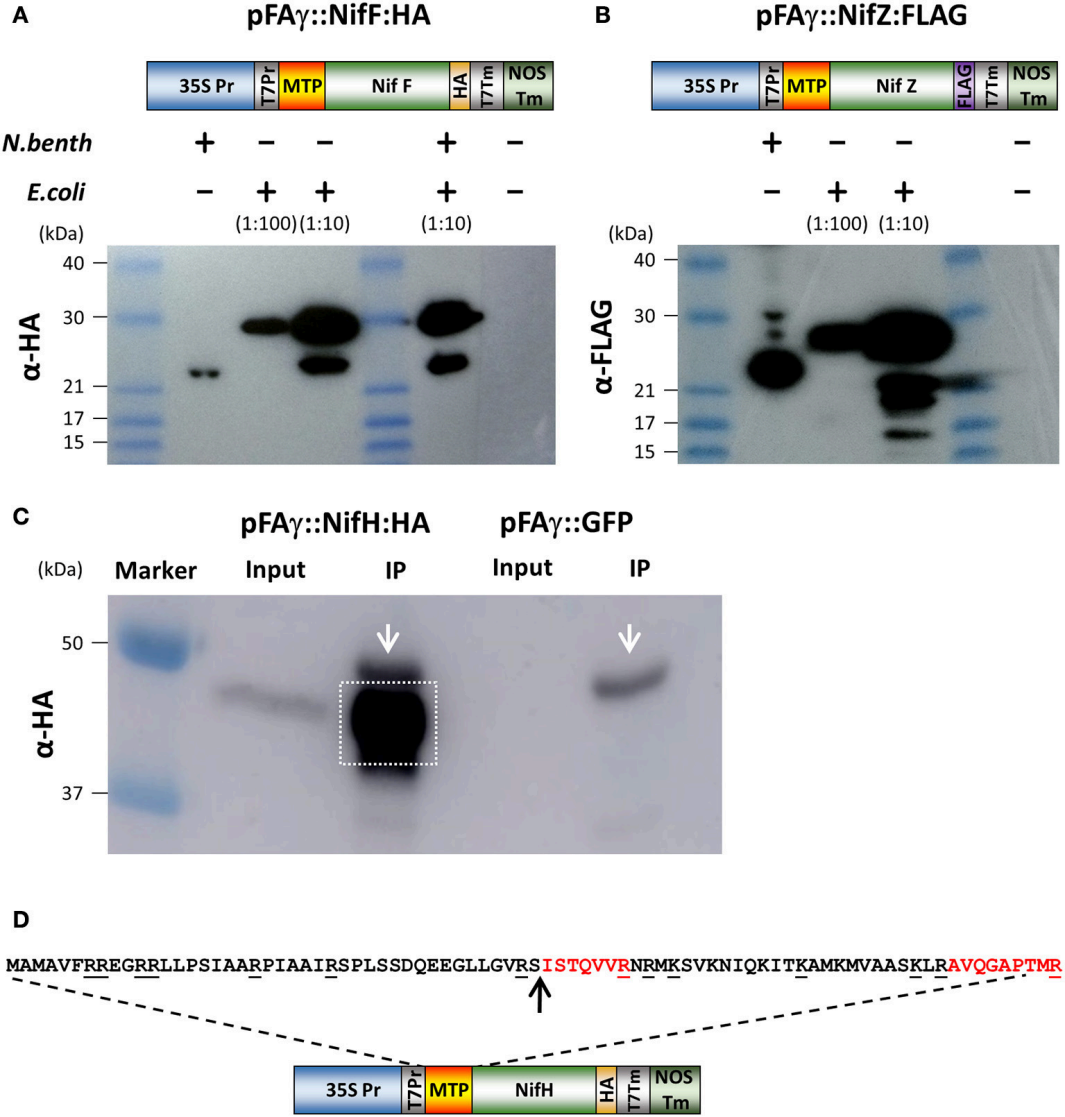
**Expression and processing of mitochondrially targeted Nif proteins in ***N. benthamiana***. (A,B)** Schematic of the pFAγ::NifF::HA and pFAγ::NifF::FLAG constructs used for *N. benthamiana* and *E. coli* expression, indicating the T7 promoter driving bacterial expression downstream of the 35S promoter for plant expression. Western blot analysis of pFAγ::NifF::HA or pFAγ::NifF::FLAG expression in *N. benthamiana* and *E. coli*. The + and − symbols above the lanes indicate the presence or absence, respectively, of *N. benthamiana* or *E. coli* protein extracts applied to the lanes; the extract dilution factors used for the bacterial extracts are indicated in brackets. **(C)** Image of Western blot for protein concentration of pFAγ::NifF::HA by anti-HA immunoprecipitation. Original protein extracts (Input) and IP eluate (IP) are shown. Background signal from the large-chain subunit of the HA antibody is marked with white arrow. The gel area excised for protein microsequencing is indicated by the white box. **(D)** Schematic of the construct used to transiently express pFAγ::NifH::HA in *N. benthamiana*. Underlined residues in the nucleotide sequence indicate sites of proteolytic cleavage (carboxyl side) by trypsin. The arrow indicates the point of cleavage by the mitochondrial processing peptidase (MPP). The peptides ISTQVVR and AVQGAPTMR were detected by mass spectrometry.

We next wanted to test if the pFAγ MTP was capable of directing bacterial nitrogenase proteins to the matrix. For this, two Nif proteins were chosen (NifF and NifZ) because their relatively small sizes would enable clear discrimination of the processed size by Western blotting. Protein-coding regions for the *K. pneumoniae* NifF and NifZ polypeptides were codon optimized for eukaryotic expression and fused to either HA or FLAG epitopes as C-terminal fusions (Figures 2A,B and Supplementary Table 1). The pFAγ MTP was fused to the N terminus of these polypeptides as for GFP above. To generate unprocessed versions of these pFAγ:: Nif proteins the same constructs were expressed in *Escherichia coli* by T7 RNA polymerase. Given that the MTP cannot be processed in bacteria which have neither mitochondria nor MPP, the difference in size between plant and bacterially- expressed proteins would enable processing to be validated by SDS-PAGE and Western blot. The Western blot revealed that the size of the polypeptides extracted from the *N. benthamiana* leaves was smaller in each case than the corresponding polypeptide produced in *E. coli* (Figures 2A,B). For each of pFAγ::NifF::HA and pFAγ::NifZ::FLAG, the polypeptides extracted from the plant cells corresponded to the sizes predicted for cleavage of the fusion polypeptides in their MTPs, whereas the polypeptides detected in *E. coli* extracts were of the expected sizes for unprocessed pFAγ::Nif fusion polypeptides. From these data we could conclude that the pFAγ MTP was capable of both directing Nif-fused polypeptides into the matrix and cleavage of the MTP by the MPP in plant cells.

Finally, we validated that the predicted MTP processing site was cleaved using mass spectrometer (MS) analysis. For this we chose NifH-HA, due to its importance as a core component of the nitrogenase enzyme complex. pFAγ::NifH::HA was expressed in plants, immunoprecipitated on HA-antibody agarose, further enriched via denaturing SDS electrophoresis (Figure 2C), and subjected to in-gel digestion with trypsin followed by tandem MS analysis of the resultant peptides. This analysis found 5 fully tryptic peptides identical to regions within NifH and a sixth semi-tryptic peptide consistent with exact cleavage of the MTP between residues 42 and 43 (Figure 2D). The tryptic peptide SISTQVVR that would been obtained from an unprocessed MTP was not observed. Instead, the most N-terminal peptide that was detected was the semi-tryptic ISTQVVR, confirmed by a complete series of y-ions in its MS/MS spectrum. These data conclusively demonstrated that the pFAγ::NifH::HA polypeptide had been cleaved at the preselected site in the MTP within the N-terminal extension by the matrix processing protease. The data implied that the pFAγ MTP contained all of signals necessary for translocation and processing in the matrix.

### Expression of individual nitrogenase proteins in the mitochondrial matrix of plants

**Figure 3:**
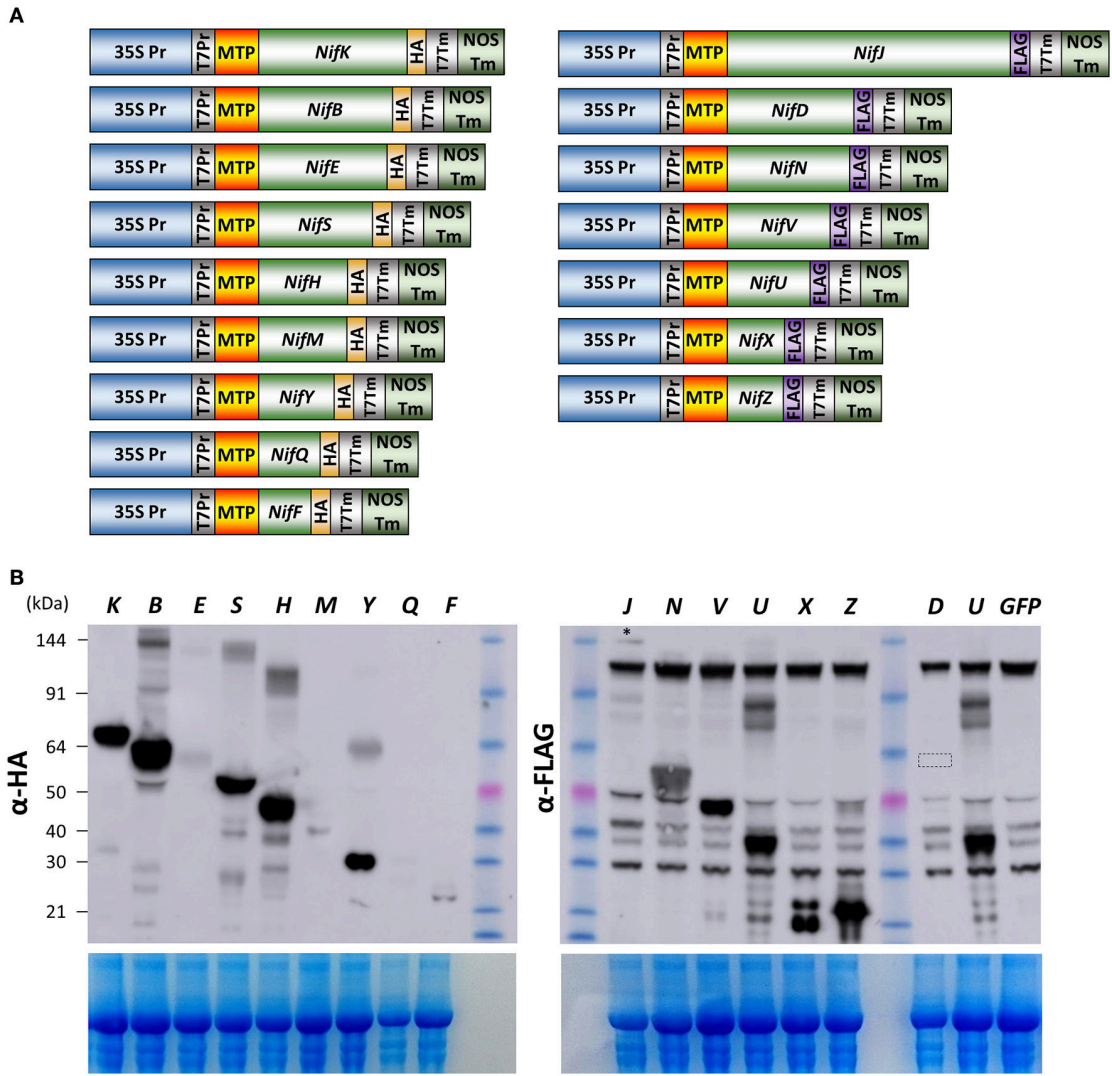
**Expression of 15 Nif proteins in the mitochondrial matrix of ***N. benthamiana***. (A)** Schematics of the constructs used for pFAγ::Nif::HA and pFAγ::Nif::FLAG expression in *N. benthamiana*. Only the Nif inserts are relatively proportional to their sequence length. **(B)** Image of Western blot probed with antibody for HA (upper left panel) or FLAG (upper right panel) after SDS-PAGE of protein extracts from *N. benthamiana* cells expressing constructs encoding pFAγ::Nif::HA or pFAγ::Nif::FLAG fusion polypeptides. The letters above the lanes (K, B, E, S etc.) indicate the Nif polypeptide included in the fusion polypeptide encoded by the genetic construct. The faint band near the top of the blot for pFAγ::NifJ::FLAG is indicated by an asterisk (^*^). A small box in the Lane “D” highlights the region of the blot where a signal for pFAγ::NiD::FLAG would be expected. The extreme right lane GFP indicates a sample extracted from a pFAγ::GFP infiltrated region as a negative control for background bands inherent to the FLAG epitope in these assays. The size of the molecular weight markers (kDa) are indicated to the left, and the same markers were used in both HA and FLAG panels. The expected sizes for processed and unprocessed proteins are shown in Supplementary Table 1. The lower panels show the corresponding gels after Coomassie staining as an indication of protein loading.

Given the success of expression, detection, and processing of GFP, NifF, NifZ, and NifH polypeptides in *N. benthamiana* mitochondria, we attempted to determine if all remaining Nif proteins required for nitrogenase biosynthesis and function could be expressed in *N. benthamiana* mitochondria. In the model diazatroph *K. pneumoniae*, 16 Nif proteins are involved in nitrogenase biosynthesis or catalysis, while four others are of unknown function or involved in transcriptional regulation (Oldroyd and Dixon, 2014). We therefore chemically synthesized eukaryotic expression codon-optimized versions of all these essential biosynthetic and catalytic *nif* genes of *K. pneumoniae*, each fused to the previously validated pFAγ MTP (Figure 3A, Supplementary Table 1). Each genetic construct encoded a fusion polypeptide having an N-terminal pFAγ MTP, then the Nif sequence, followed by a C-terminal extension comprising either an HA or FLAG epitope for detection by the appropriate antibody. For plant expression, each construct included the 35S promoter and nopaline synthase (nos) 3′ transcription terminator regions flanking the protein coding region.

*A. tumefaciens* cells containing each of the 16 genetic constructs were separately infiltrated into *N. benthamiana* leaves and, 4 days later, protein extracts prepared and analyzed by SDS-PAGE and Western blotting as before. For each of the constructs encoding HA-tagged pFAγ-Nif polypeptides, bands were detected on the Western blots which were approximately the size as predicted for the matrix-processed polypeptide (Figure 3B). Protein abundance varied among the HA-tagged polypeptides, with the easiest to detect being the NifB, NifH, NifK, NifS, and NifY fusion polypeptides. The NifF, NifE, and NifM polypeptides were present at lower levels, whereas detection of the NifQ polypeptide required a longer exposure of the blot to be visible (Supplementary Figure 1). Interestingly, additional, higher-molecular weight bands were detected for the infiltrations with the NifB, NifS, NifH, and NifY constructs that were size-specific to each individual construct. These additional bands were approximately twice the molecular weight of the primary band, suggesting that these polypeptides were dimerising despite the denaturing conditions during gel electrophoresis. It has been reported the NifB, NifS, and NifH proteins function as homo-dimers in bacteria (Yuvaniyama et al., 2000; Rubio and Ludden, 2008).

**Figure 4:**
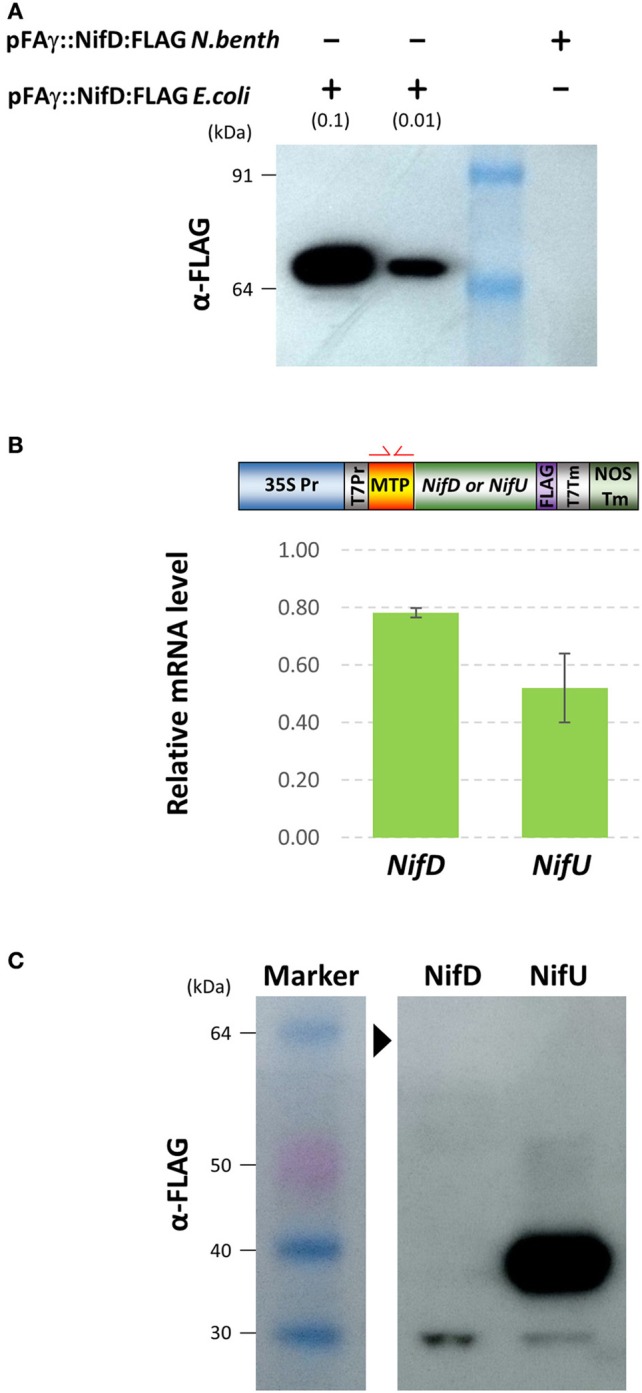
**Expression of MTP-NifD-FLAG is undetectable in ***N.benthamiana*** despite high relative mRNA expression. (A)** Western blot of anti-FLAG for pFAγ::NifD::FLAG transformed *E. coli* or *N. benthamiana*. The blot was probed with the antibody against the FLAG epitope. The molecular weights of the markers in the first lane are indicated. The + and − symbols above the lanes indicate the presence or absence, respectively, of *N. benthamiana* or *E. coli* protein extracts applied to the lanes; the extract dilution factors used for the bacterial extracts are indicated in brackets. **(B)** qRT-PCR analysis of *pFA*γ*::Nif::FLAG* transgene expression. Schematic above shows the primers annealing in the MTP region. Expression values were normalized to GADPH, with measurements being the average of three replicates and error bars representing the standard error of the mean. **(C)** Image of Western blot using anti-FLAG for protein extracts prepared from *N. benthamiana* leaf samples 4 days after infiltration with either pFAγ::NifD::FLAG or pFAγ::NifU::FLAG. Black arrowhead indicates the expected position for a pFAγ::NifD::FLAG band. Protein marker sizes are indicated on left hand side of image.

FLAG-tagged pFAγ::Nif::FLAG fusion polypeptides were observed in the Western blots for each of the constructs including the NifJ, NifN, NifV, NifU, NifX, and NifZ sequences (Figure 3B). The FLAG antibody yielded more background bands from the *N. benthamiana* extracts than the HA antibody. Nevertheless, the results were similar to those for the HA-tagged proteins. Considerable variation was seen in signal intensity for the different pFAγ::Nif::FLAG fusion polypeptides. An additional, higher-molecular-weight band was observed that was specific for the NifU construct (Figure 3B). The pFAγ::NifX::FLAG construct also yielded an additional, smaller band of greater intensity than expected for the predicted, processed molecular weight. Despite numerous replications of the infiltrations, we were unable to detect any specific bands for the pFAγ::NifD::FLAG construct in these plant assays, even though expression of the same genetic construct in *E. coli* readily yielded a visible band of the expected molecular weight (Figure 4A). The result from the bacterial extracts confirmed that the genetic construct containing pFAγ::NifD::FLAG was translationally competent.

### Improved MTP-NifD production in *N. benthamiana*

Given the key role of NifD in the catalytic activity of nitrogenase (Eady et al., 1972), we tried to identify the reason for the lack of NifD fusion polypeptide production and tested several approaches to improve its abundance in plant assays. Firstly, we tested whether the lack of MTP-NifD fusion polypeptide production/accumulation could be attributed to low transgene transcription or mRNA instability, by measuring the mRNA expression level. To do this, the level of mRNA in the infiltrated *N. benthamiana* cells from pFAγ::NifD::FLAG was first measured by qRT-PCR and compared to the level of mRNA transcribed from the construct encoding pFAγ::NifU::FLAG in *N. benthamiana* cells (Figure 4B). This second construct was used as a control since it provided high levels of polypeptide production in the plant cells, as described above (Figure 4C). In order to remove any bias in amplification efficiency, oligonucleotide primers were used which annealed within the pFAγ MTP region shared by both *nif* fusion genes (illustrated in Figure 4B, Supplementary Table 2). The results from the qRT-PCR assay showed that the level of *pFA*γ*::nifD::FLAG* mRNA was actually slightly higher, although not significantly (*P* = 0.0683), than the level of *pFA*γ*::nifU::FLAG* mRNA. Secondly, cDNA was synthesized from the plant-produced mRNA transcribed from *pFA*γ*::nifD::FLAG*, cloned and sequenced, and its nucleotide sequence proved to be base perfect. Therefore, the lack of accumulation of the pFAγ::NifD::FLAG fusion polypeptide was not due to low mRNA expression or instability. These experiments also showed that the T-DNA containing and encoding pFAγ::NifD::FLAG was fully functional and that the 35S promoter in that construct was likewise functional. Therefore, the inability to detect the MTP-NifD fusion polypeptide in *N. benthamiana* cells was not due to any lesions in expression of the mRNA.

**Figure 5:**
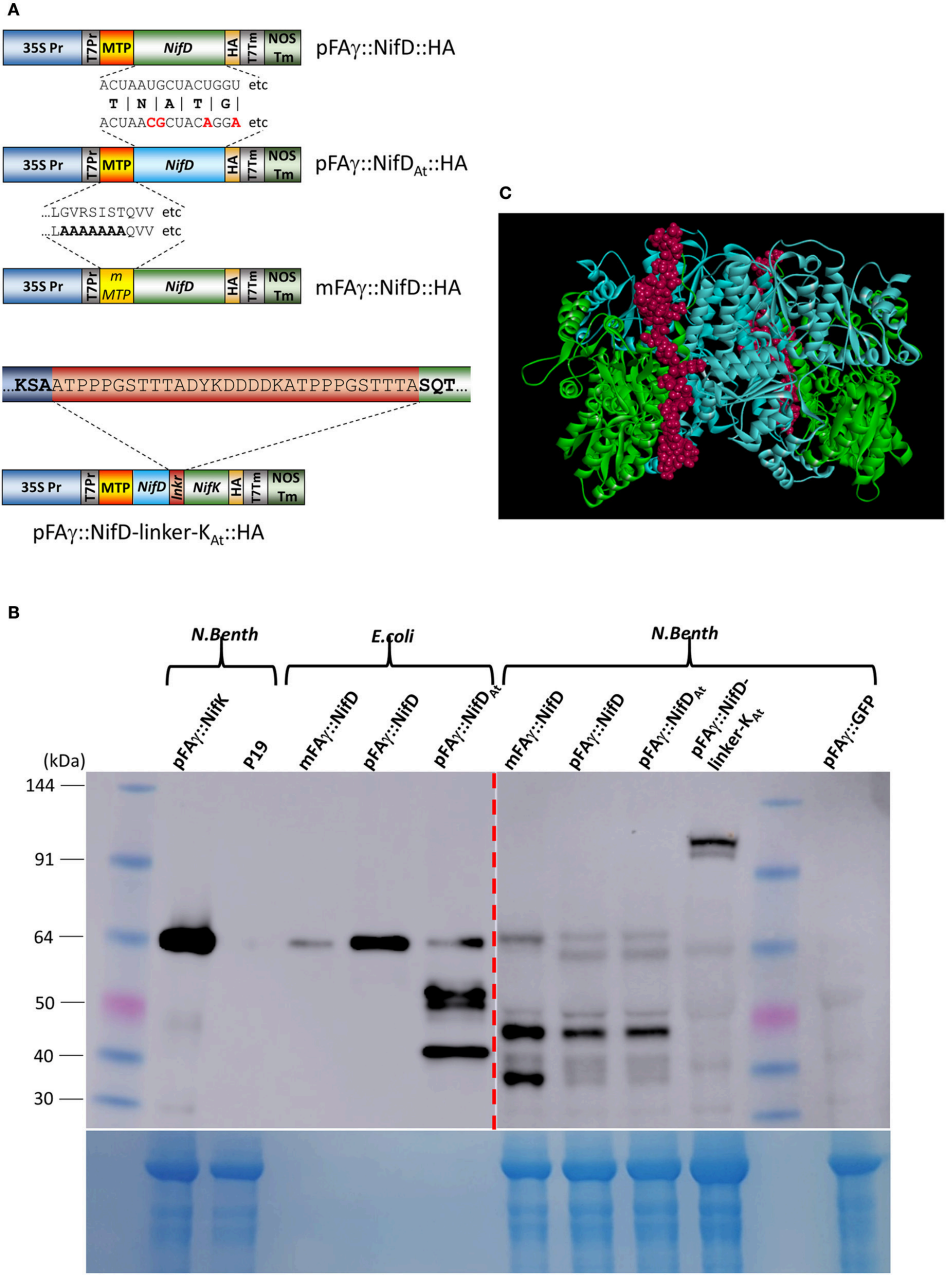
**Improved expression of NifD polypeptide fusions in ***N. benthamiana***. (A)** Schematics of the various NifD constructs used for expression analysis. Examples of RNA or protein sequence differences are shown; protein sequences for pFAγ::NifD::HA and pFAγ::NifD_At_::HA are identical, but the codon usages for the NifD coding regions vary, only the first 18 nucleotides with differences in bold red are shown for space. mMTP indicates the region encompassing the mFAγ MTP, which contains alanine substitutions identical to mFAγ::GFP for disruption of mitochondrial matrix translocation. For pFAγ::NifD-linker-NifK::HA, the entire linker is shown in the red bar, flanked by the NifD (blue) and NifK (green) sequences. **(B)** Western blot analysis of protein extracts from cells expressing Nif polypeptide fusions and probed with anti-HA. Proteins extracts were prepared from either *E. coli* or *N. benthamiana* indicated by bracketed areas above the blot. The image output levels were adjusted on the right hand side of the blot (shown by red dashed arrow) to prevent oversaturation by *E. coli* bands (original image shown in Supplementary Figure 2). The corresponding Coomassie-stained gel is shown underneath as an indication of protein loading, noting that bacterial extracts have a different total protein profile from that in leaf extracts. **(C)** *In silico* representation of the structure of NifD-linker-NifK shown as the α_2_β_2_ heteroteramer. The blue residues are NifD, green residues NifK and the linker displayed as red.

As transcription of the gene encoding pFAγ::NifD::FLAG and mRNA accumulation was clearly not limiting MTP-NifD production, several modifications were made to the genetic construct in attempts to improve protein accumulation (Figure 5A). Firstly, the possibility was tested that the presence of the FLAG epitope in the C-terminal extension was causing either lack of production or instability of the NifD fusion polypeptide. For this, a construct was designed in which the FLAG epitope was substituted with an HA epitope, designated pFAγ::NifD::HA. The HA epitope had allowed for accumulation and detection of 9 other MTP-Nif proteins (Figure 3B). Secondly, the codon usage of the NifD::HA open reading frame was modified in an attempt to determine whether a different mRNA sequence might improve translation efficiency. For this, an *A. thaliana* codon optimization algorithm was utilized (Graf et al., 2009), whereas the previous construct used a *H. sapien* algorithm (Graf et al., 2009). This construct was designated pFAγ::NifD_At_::HA, and encoded an identical polypetide to pFAγ::NifD::HA. Additionally, a genetic construct was made encoding a version of the NifD fusion polypeptide with the mutated mFAγ N-terminal extension rather than pFAγ (mFAγ::NifD::HA) as described earlier (Figure 1A). This construct was made in order to test whether mitochondrial targeting and/or processing, if they occurred, were at least partially responsible for the lack of NifD fusion polypeptide production.

*N. benthamiana* leaves were infiltrated with *A. tumefaciens* containing these constructs and protein extracts prepared from the infiltrated tissues and analyzed. Encouragingly, the Western blot showed HA-containing bands of the molecular weights expected for both matrix-processed and unprocessed NifD fusion polypeptides when either pFAγ::NifD::HA or pFAγ::NifD_At_::HA constructs were introduced (Figure 5B). Introduction of mFAγ::NifD::HA yielded only the larger (unprocessed) fusion polypeptide, showing that as for mFAγ::GFP (Figure 1A), this modification of the pFAγ MTP disrupts matrix processing. Matrix processing was further verified by comparing the position of the bands with that produced from mFAγ::NifD::HA and the bacterially produced polypeptides (Figure 5B). The observation of two bands, corresponding in size to processed and unprocessed forms of pFAγ::NifD::HA or pFAγ::NifD_At_::HA indicated that processing of this MTP-NifD fusion polypeptide was not as efficient as for the other MTP-Nif fusion polypeptides as described above. The observed level of the pFAγ::NifD::HA or pFAγ::NifD_At_::HA polypeptides were still much lower than the level of the pFAγ::NifK::HA fusion polypeptide used as a positive control, despite the same expression construct design and expression conditions. Furthermore, for each of the three modified NifD::HA constructs, additional bands of lower molecular weight were observed on the Western blots, some of which were specific to a particular pFAγ::NifD::HA version. For example, an intense band at about ~48 kDa was present for all of the modified NifD::HA constructs, but a different, intense band at about 40 kDa appeared unique when mFAγ::NifD::HA was introduced. As these bands were not present for either pFAγ::NifK::HA or GFP controls, they could represent NifD::HA degradation products or possibly the product of alternative transcription or translation initiation signals.

### Design and expression of a translational fusion between NifD and NifK

In diazatrophic bacteria the abundance of NifD and NifK are almost equal (Poza-Carrion et al., 2014) and both are found in crystal structures of nitrogenase in a 1:1 ratio as a heterodimer (Schmid et al., 2002). However, it was clear from the above analysis that NifK accumulation was significantly higher than NifD. Given that NifD and NifK form the catalytic unit of nitrogenase, functional activity in plants would likely benefit from a higher level of NifD and a stoichiometric 1:1 NifD:NifK ratio. We therefore designed a translational fusion between NifD and NifK to link the expression of these proteins, as similar recombinant fusion strategies have been shown to improve accumulation (Hondred et al., 1999). This approach required the design of an amino acid linker joining the NifD and NifK units whilst still allowing the proper protein folding of the entire fused D-K polypeptide, as follows. A homology model of the FeMoco protein (NifD and K) α_2_β_2_ hetero-tetramer from *K. pneumoniae* was constructed using the crystal structure of the FeMoco protein complex from *Azotobacter vinelandii* (PDB ID: 1FP4; Schmid et al., 2002) as a template. In the structural model for the *K. pneumoniae* D_2_K_2_ hetero-tetramer, the C-terminus of each NifD subunit was approximately 47 Å from the N-terminus of the NifK partner. Therefore, an unstructured and flexible amino acid linker of 30 residues was designed to span this 47 Å length (Figure 5A; see Section Materials and Methods for full details of the *in silico* design protocol). The predicted structure of the NifD-linker-NifK composite polypeptide, including its 30-amino acid linker, was examined, showing that addition of the linker allowed the full fusion polypeptide to fold into a structure that mimicked the native MoFe complex (Figure 5C). Finally a HA epitope tag was appended to the C-terminus of NifK (as previously for singly expressed NifK) to enable discrimination of the fusion protein with HA antibody, here termed pFAγ::NifD-linker-NifK::HA (Figure 5A). When this vector was introduced into *N. benthamiana* cells and protein extracts examined by Western blotting, two specific bands were detected in the size range predicted for the polypeptide at ~120 kDa. The closeness of the two bands suggested that both unprocessed and processed forms for the MTP presequence were present. The upper band was of greater intensity than the lower band, suggesting that processing occurred only partially. Nevertheless, the level of accumulation of the pFAγ::NifD-linker-NifK::HA polypeptide was greater than for pFAγ::NifD::HA, but much less than for pFAγ::NifK::HA.

### Stacking multiple nitrogenase proteins in the mitochondrial matrix of plants

**Figure 6:**
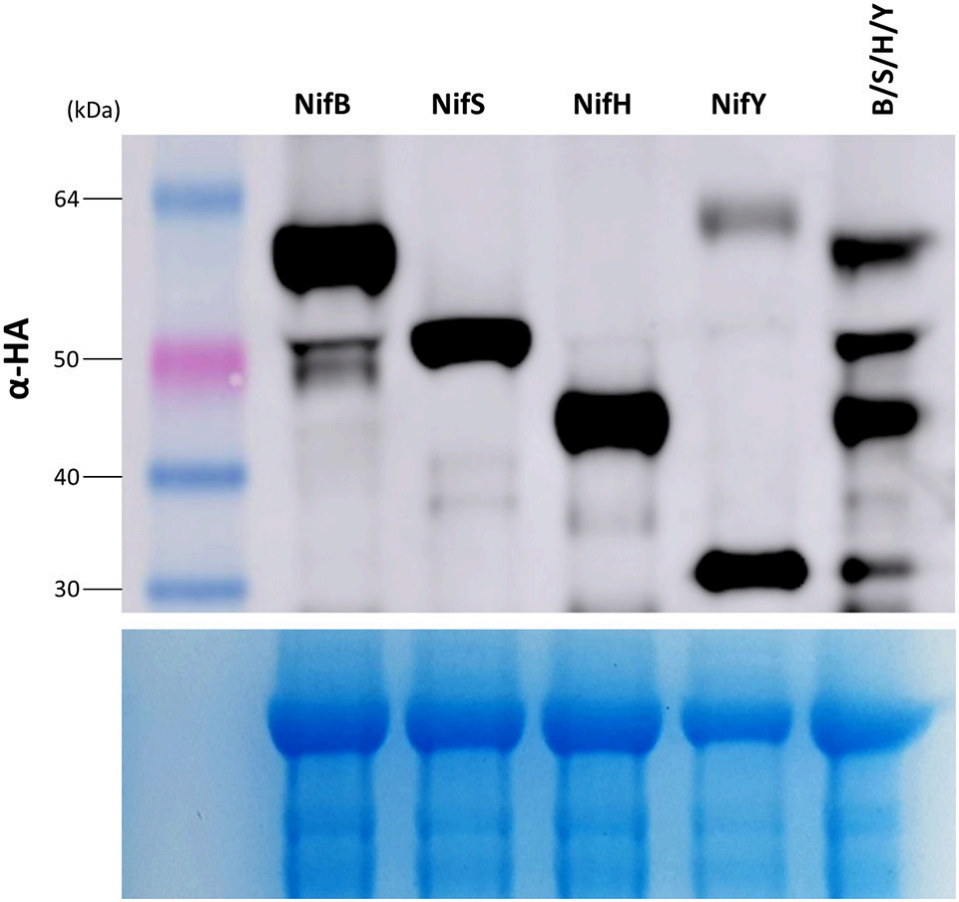
**Stacking of multiple Nif proteins in the mitochondrial matrix of ***N. benthamiana*****. **(Upper panel)** Western blot of protein extracts for constructs expressing pFAγ::Nif::HA either singly (NifB, NifS, NifH, NifY), or as a combination of the same four individual Agrobacterium cultures infiltrated in an equimolar mixture of NifB, S, H, Y. Sizes of markers are indicated on left. The corresponding Coomassie-stained gel is shown in the **(lower panel)**, indicating even loading across all lanes.

As nitrogenase activity requires the concerted action of many Nif proteins, it is anticipated that functional nitrogenase reconstitution in plants will require many of the Nif proteins utilized by diazotrophic bacteria for biosynthesis and function. We therefore wanted to determine if multiple Nif proteins could be expressed in *N. benthamiana* using the pFAγ MTP. To test this concept, genetic constructs encoding four Nif fusion polypeptides of different sizes were chosen that would enable the resultant polypeptides to be identified by Western blot analysis, namely the constructs encoding pFAγ::NifB::HA, pFAγ::NifS::HA, pFAγ::NifH::HA, and pFAγ::NifY::HA. The four *A. tumefaciens* cultures transformed with these constructs were mixed in equal amounts and the mixture was then infiltrated into *N. benthamiana* leaves. The accumulated polypeptide levels in this four construct combination were compared to infiltrations with single constructs. It was observed that each polypeptide was more abundant when expressed from a single construct than from the four gene combination (Figure 6). Nevertheless, all four Nif fusion polypeptides were readily detected in the protein extracts from the gene combination, and the molecular weight observed for each polypeptide was identical for the individual and combination infiltrations. This showed that combinations of Nif fusion polypeptides could be produced in the plant cells with the desired targeting and processing of each Nif fusion polypeptide to the mitochondria.

## Discussion

The model diazotroph *K. pneumoniae* requires 16 unique proteins for the biosynthesis and catalytic function of nitrogenase. We have established that this set can be individually expressed as MTP:Nif polypeptide fusions in the mitochondrial matrix of plants, a subcellular location potentially accommodating for nitrogenase function. This is the first practical demonstration of the feasibility of such an approach in plants, and represents important progress toward the aim of engineering plants with nitrogenase activity.

In this study we chose to target Nif proteins to the matrix, primarily because endogenous oxygen consuming enzymes may enable nitrogenase function in a similar manner to the respiratrory protection provided by aerobic diazatrophs (Rey and Maier, 1997). A targeting peptide previously demonstrated to be capable of directing GFP to the *Arabidopsis* matrix was used for this purpose (Lee et al., 2012), and was relatively long to assist detection of the processed protein (Figure 1). From our analysis, we are confident the chosen MTP targeted most, if not all nitrogenase proteins to the matrix. We make this claim based on several lines of evidence. Firstly, the sizes observed for *N. benthamiana* expressed Nif proteins were consistent with the expected size resulting from matrix peptidase processing (Figures 2, 3). This was also reflected by the difference in size observed between bacterial and plant mitochondrially expressed Nif proteins (NifF and NifZ; Figures 2, 3). Additionally when we mutated the MTP, rendering it incapable of being processed by the mitochondrial import machinery, a larger polypeptide was observed for both NifD and GFP fusion polypeptides, consistent with the difference in size between processed and unprocessed polypeptides (Figures 1, 4). Finally and conclusively, mass spectrometry determined that pFAγ::NifH::HA was cleaved between residues 42–43 of the MTP as expected for specific processing in the matrix (Figure 2).

The presence of the pFAγ MTP did not always lead to complete processing of Nif proteins. In some instances (pFAγ::NifX::FLAG, pFAγ::Nif::HA, and pFAγ::NifD-linker-K::HA), both processed and unprocessed Nif proteins were observed (Figures 3, 5). Considering there is no consensus sequence for MTPs, and internal protein sequences can influence mitochondrial targeting (Becker et al., 2012), it is perhaps not surprising that we found differences in processing efficiency amongst the Nif proteins. Although, the chosen MTP appears well suited for the majority of Nif protiens, other targeting peptides may need to be explored for efficient processing of NifD, NifD-linker-K, and NifX. Furthermore, additional studies are required to address the consequences of adding MTP-related residues to the N-terminal of Nif proteins with regard to overall function.

Despite use of the strong, constitutive 35S promoter, a remarkable degree of variability in protein abundance was observed for the various Nif proteins (Figures 3, 5). Whilst the expression levels of different Nif genes are exquisitely optimized in diazatrophic bacteria such as *K. pneumoniae* (Temme et al., 2012), it is difficult to predict what the specific requirements will be in *N. benthamiana*. In this exploratory study, we have focused on finding expression and evidence of processing of each Nif protein, but modifying the expression level of each Nif protein to match bacterial profiles may be required for optimized activity.

Intriguingly, additional higher-molecular-weight protein bands were also found for several of the individual Nif proteins expressed in plants, despite denaturing conditions (see Section Materials and Methods). This was most apparent for Nif B, S, H, and Y; these fainter bands were approximately double the dominant band size, suggestive of a homo-dimerization (Figure 3). In bacteria NifB, S, H, and U function as homodimers in (Yuvaniyama et al., 2000; Rubio and Ludden, 2008). Possibly these additional bands may be higher order structures representative of bacterial functional equivalents. Alternatively, endogenous proteins may interact strongly with transgenic Nif proteins. Shorter peptide fragments of some Nif proteins were also detected via the epitope at the C-terminal of the protein. These fragments may arise due to errant transcription, translation, or protease decay of the mature Nif protein to a shorter size.

Of all the Nif proteins, the essential component required for nitrogenase catalytic activity, NifD, was the most difficult to express as a pFAγ fusion (Figures 3, 4). The low levels of pFAγ::NifD protein were in contrast to the high levels of *pFA*γ*::NifD* RNA, suggesting translation rates or protein stability were limiting pFAγ::NifD protein abundance. Given the critical importance of NifD in catalysis, its requirement to be highly expressed in bacteria (Poza-Carrion et al., 2014), and in an equimolar ratio with NifK, we designed a translational fusion of these two key proteins and found that NifD abundance could be enhanced through this strategy (Figure 5). Given that in bacteria a functional NifD protein requires the interaction of several Nifs (Hu and Ribbe, 2013) the enhancement of NifD we have found by co-translation with NifK is encouraging for future studies, where further Nif precursor proteins will need to be co-expressed.

This NifD-linker-K fusion also possessed the advantage of being expressed as a single protein, allowing translation of both subunits at the ideal 1:1 ratio, therefore mimicking the stoichiometry of the native heterotetramer. Furthermore, the linker itself was designed to allow sufficient flexibility for the two subunits to form the correct α_2_β_2_ heterotetrameric structure required for catalysis. An earlier report demonstrated that a NifD-K translational fusion without such a flexible linker can impart a limited degree of function (Suh et al., 2003; Lahiri et al., 2005). Therefore, we anticipate our version of NifD-linker-K will at least functionally substitute for individual NifD and NifK expression, possibly with greater efficacy than previously demonstrated. This hypothesis could be tested via a complementation test for nitrogenase activity in bacterial mutants for NifD and NifK.

Re-engineering nitrogenase into plants will ultimately require many Nif proteins to be co-expressed. Here we show that NifB, NifS, NifH, and NifY are at least capable of co-expression within the same tissue (Figure 6). Whilst experiments by others have demonstrated that different proteins with identical MTPs can be targeted to the same mitochondria (Yang et al., 2010), additional experiments will be required to confirm if all these Nifs accumulate together within the same mitochondria. Furthermore, increasing the number of *nif* genes for transient assays was found to dilute their individual expression, therefore future strategies will need to explore methods to overcome this issue. In this regard, protein fusions such as used here for NifD-linker-K, and multigene cassettes that have been used successfully in previous gene stacking experiments (Naim et al., 2012; Petrie et al., 2014), may be helpful.

Encouragingly, there is emerging evidence that a smaller subset of Nif proteins may be required to achieve functional nitrogenase reconstitution in plant mitochondria. In diazotrophs, genetic approaches have shown that a functional Fe subunit requires 4 proteins, NifS, NifU, NifM, and NifH. Remarkably only NifH and NifM were required to enable *ex vivo* Fe protein function from yeast mitochondria (López-Torrejón et al., 2016). Similarly, *ex vivo* Fe protein activity was achieved by expression of NifH and NifM via transformation of the chloroplast genome (Ivleva et al., 2016). These two reports indicate that in eukaryotic subcellular compartments a biochemically active Fe subunit can be assembled without the requirement for NifS or NifU transgenes.

These same studies also provide the first experimental evidence that subcellular organelles may be sufficient to support nitrogenase activity, as the Fe subunit is highly sensitive to oxygen. In plant chloroplasts *ex vivo* Fe protein activity required the lowering of ambient oxygen conditions (Ivleva et al., 2016). By contrast, aerobically grown yeast expressing matrix targeted NifH and NifM were capable of producing a functional Fe protein (López-Torrejón et al., 2016). Taken together, these two studies indicate subcellular plant organelles may support nitrogenase activity, and the matrix, as an oxygen consuming environment may be the most preferable location.

Although, the MoFe protein is less oxygen sensitive than the Fe subunit (Eady et al., 1972), its biogenesis is undoubtedly more complex. Here we demonstrate that a complete repertoire of Nif proteins can be expressed in plants, including those required for MoFe subunit assembly, in a subcellular location that is potentially supportive of nitrogenase function. This study adds to the experimental evidence that transgenic plants can be generated to be self-sufficient for bioavailable nitrogen in the future.

## Materials and methods

### Construction of vectors

Vector pCW441 was designed as dual purpose bacterial and plant expression construct. A region of the pET14b vector (Novogene) was used to design a region of DNA encompassing the T7 promoter, a simple multiple cloning site (*Not*I and *Asc*I) and the T7 terminator, and this region was chemically synthesized (Geneart) and ligated into a T-DNA-based plant expression vector, pORE1 (Coutu et al., 2007) in between the 35S promoter and the NOS terminator regions, generating pCW441. This vector is stable in *E. coli*, and can produce proteins via the standard T7 polymerase promoter system (Studier and Moffatt, 1986). The 240 bp sequence incorporating the 77 amino acid MTP of the F1-ATPase γ subunit (Lee et al., 2012) was chemically synthesized with a *Not*I and *Asc*I flanking region permitting ligation into pCW441, generating pRA1. *Klebsiella oxytcoa* Nif genes were codon optimized for eukaryotic expression and commercially synthesized (Geneart). Nif gene sequences were flanked by AscI sites for subcloning and contained either HA or FLAG tags as C terminal fusions and designed to allow a translational fusion with the N-terminal MTP. GFP sequence was derived from S65T GFP sequence (Ormö et al., 1996). The full compendium of constructs used in this study are found in Supplementary Table 1.

### Growth of *Agrobacterium* and *N. benthamiana* infiltrations

Plant growth and Agrobacterium infiltrations were carried out as described by Naim et al. (2012).

### Protein extractions and western blot analysis

Infiltrated *N. benthamina* leaf proteins were extracted by grinding an ~2 × 2 cm leaf disc in liquid nitrogen then transferring the powder to 300 μL of buffer comprised of 125 mM Tris-HCL pH 6.8, 4% SDS, 20% glycerol, 60 mM DTT. Samples were heated at 95°C for 3 min before centrifugation at 12,000 g for 2 min. Protein samples (20 μL) were separated by SDS-PAGE (NuPAGE Bis Tris 4–12%, Thermofisher.com) at 200 V for 1 h. Proteins were transferred to PVDF membranes using an iblot system (Thermofisher). After blotting gels were Coomassie stained for 1 h then rinsed in water for visualization of remaining protein. Membranes were blocked overnight in TBST + 5% skim milk powder at 4°C. Anti-HA, and anti-FLAG were purchased from Sigma, anti-GFP was a gift from Leila Blackman (ANU). Antibodies were added at 1:5,000 in TBST with 5% skim milk powder and incubated for 2 h. Membranes were washed for 3 × 20 min with TBST and the secondary antibody [Immun-Star Goat Anti-Mouse (GAM)-HRP conjugate] (Biorad) was added at 1:5,000 in TBST + 5% skim milk for 1 h, followed by 3 × 15 min TBST washes. For secondary antibody detection Amersham ECL reagent was used and membranes were developed either with an X-ray developer or on an Amersham imager (Amerhsam).

### Protoplast preparation, mitochondria staining, and laser-scanning confocal microscopy

Protoplasts from infiltrated and untransformed *N. benthamiana* leaves were prepared as described before (Rolland et al., 2016). Mitochondria were stained for 10–20 min using a 100 nM solution of MitoTracker Red CMXRos (ThermoFisher Scientific). Protoplasts were then imaged using an upright Leica SP8 laser-scanning confocal microscope with a 40x water immersion objective (NA = [1.1]). GFP was excited at 488 nm and emission was recorded at 495–520 nm. In the same track, MitoTracker Red CMXRos was excited at 580 nm and emission was recorded at 595–620 nm. In a separate track, chloroplasts were excited at 633 nm and emission was recorded at 650–690 nm.

### RNA analysis

RNA was extracted from infiltrated *N. benthamina* leaves by grinding an ~2 × 2 cm leaf disc in liquid nitrogen then transferring the powder to 500 μL of Trizol (Life Technologies) buffer for RNA purification. RNA expression analysis was as described previously (Allen et al., 2010) and *N. bentahamiana* GADPH was used to normalize gene expression. Primers are described in Supplementary Table 2.

### Design of a NifD-Linker-NifK translational fusion

A homology model of the NifD-K α_2_β_2_ heterotetramer from *K. pneumoniae* was constructed using the crystal structure of the NifD-K complex from *A. vinelandii* (PDB ID: 1FP4) as a template. The C-terminus of NifD was ~47 Å from the N-terminus of its NifK partner so a linker of appropriate length was designed to connect the two units. The linker was 30 residues in length consisting of an 11-residue section from a known unstructured linker region from *Hypocrea jecorina* cellobiohydrolase II (Accession no. AAG39980.1) with the final arginine replaced by an alanine, followed by an 8-residue FLAG-tag and finally by another copy of the 11-residue unstructured linker sequence with the arginine replaced by an alanine. A geometry optimization and equilibration at constant pressure was carried out on the tetramer using an octahedral TIP3P water box with minimum boundary distance from the solute of 10.0 Å (without the inclusion of any of the metal centers). This calculation was carried out using Amber 12 (Case et al., 2012) employing the ff99SB force field at 298 K over 5 ns in total using 2 fs time steps and SHAKE constraints. The final NifD-linker-NifK sequence is found in Supplementary Table 1.

### Tandem mass spectrometer analysis

Infiltrated *N. benthamiana* tissue was ground in liquid N_2_ and processed using a Retsch tissuelyser in 2 mL Eppendorf tubes in 50 mM Tris-HCL pH7.5, 1 mM EDTA, 150 mM NaCl, 0.2% (w/v) SDS, 10% (v/v) glycerol, 5 mM DTT, 0.5 mM PMSF, and 1% (v/v) protease inhibitor cocktail for plants (Sigma). Protein extracts were cleared by centrifugation and used as input for overnight incubation with monoclonal anti-HA conjugated agarose beads (Sigma). Unbound proteins were removed by a series of washes with 150 mM Tris-HCL pH 7.5, 5 mM EDTA, 150 mM NaCl, 0.1% (w/v) Triton X-100, 5% (v/v) glycerol, 5 mM DTT, 0.5 mM PMSF, and 1% protease inhibitor cocktail for plants (Sigma). Bound proteins were eluted by incubating beads in Laemmli buffer at 95°C for 10 min. Input and IP protein samples were separated by SDS-PAGE and the area of gel determined to contain MTP-NifH-HA was determined by Western analysis upon a duplicated sample. From the replicate gel, the corresponding region was excised for in-gel tryptic digestion and tandem mass spectrometer analysis as previously described using an Agilent Chip Cube system coupled to an Agilent Q-TOF 6550 mass spectrometer (Campbell et al., 2014). Mass spectra derived from tryptic peptides from common contaminants such as the added trypsin and human keratin were identified before the remaining mass spectral data were used to search against a database containing all protein sequences from *Nicotiana* species from the NCBInr database (10/3/2015) plus the HA sequence using Spectrum Mill software (Agilent Rev B.04.01.141 SP1) with a precursor mass tolerance of 15 ppm, product mass tolerance of 50 ppm, default Q-TOF scoring, and stringent default “autovalidation” settings. Modification of cysteine residues by acrylamide was a required modification and oxidation of methionine was allowed as a variable modification. Initially, tryptic cleavage was required and up to two missed cleavages were allowed. After validating peptide matches, the search was repeated with the remaining unmatched spectra allowing non-tryptic cleavage.

## Author contributions

RA Conceived designed and performed experiments, wrote the paper. CW Conceived designed and performed experiments, wrote the paper. VR Designed and performed experiments, helped with writing the paper. PC Performed experiments, helped with writing the paper. KT Designed and performed experiments, helped with writing the paper. AW Designed and performed experiments, helped with writing the paper. SS Helped with experimental design and writing the paper.

### Conflict of interest statement

This work is subject to a patent by CSIRO in which RA, KT, AW and CW are inventors. The other authors declare that the research was conducted in the absence of any commercial or financial relationships that could be construed as a potential conflict of interest.

## References

[B1] Allen R. S. Li J. Alonso-Peral M. M. White R. G. Gubler F. Millar A. A. (2010). MicroR159 regulation of most conserved targets in Arabidopsis has negligible phenotypic effects. Silence 1, 18–18. 10.1186/1758-907X-1-18 21029441 PMC2988730

[B2] Balk J. Pilon M. (2011). Ancient and essential: the assembly of iron-sulfur clusters in plants. Trends Plant Sci. 16, 218–226. 10.1016/j.tplants.2010.12.006 21257336

[B3] Becker T. Böttinger L. Pfanner N. (2012). Mitochondrial protein import: from transport pathways to an integrated network. Trends Biochem. Sci. 37, 85–91. 10.1016/j.tibs.2011.11.004 22178138

[B4] Campbell P. M. Trueman H. E. Zhang Q. Kojima K. Kameda T. Sutherland T. D. (2014). Cross-linking in the silks of bees, ants and hornets. Insect Biochem. Mol. Biol. 48, 40–50. 10.1016/j.ibmb.2014.02.009 24607851

[B5] Case D. A. Darden T. A. Cheatham T. E. Simmerling C. L. Wang J. Duke R. E. (2012). AMBER 12. San Francisco, CA: University of California.

[B6] Coutu C. Brandle J. Brown D. Brown K. Miki B. Simmonds J.. (2007). pORE: a modular binary vector series suited for both monocot and dicot plant transformation. Transgenic Res. 16, 771–781. 10.1007/s11248-007-9066-2 17273915

[B7] Cui S. Shi Y. Groffman P. M. Schlesinger W. H. Zhu Y.-G. (2013). Centennial-scale analysis of the creation and fate of reactive nitrogen in China (1910–2010). Proc. Natl. Acad. Sci. U.S.A. 110, 2052–2057. 10.1073/pnas.1221638110 23341613 PMC3568337

[B8] Curatti L. Rubio L. M. (2014). Challenges to develop nitrogen-fixing cereals by direct nif-gene transfer. Plant Sci. 225, 130–137. 10.1016/j.plantsci.2014.06.003 25017168

[B9] De'ath G. Fabricius K. E. Sweatman H. Puotinen M. (2012). The 27-year decline of coral cover on the Great Barrier Reef and its causes. Proc. Natl. Acad. Sci. U.S.A. 109, 17995–17999. 10.1073/pnas.1208909109 23027961 PMC3497744

[B10] de Bruijn F. J. (ed.). (2015). The quest for biological nitrogen fixation in cereals: a perspective and prospective, in Biological Nitrogen Fixation (Hoboken, NJ: John Wiley & Sons, Inc.), 1087–1101.

[B11] Eady R. R. Smith B. E. Cook K. A. Postgate J. R. (1972). Nitrogenase of *Klebsiella pneumoniae*. Purification and properties of the component proteins. Biochem. J. 128, 655–675. 10.1042/bj1280655 4344006 PMC1173817

[B12] Geddes B. A. Ryu M. H. Mus F. Costas A. G. Peters J. W. Voigt C. A.. (2015). Use of plant colonizing bacteria as chassis for transfer of N-2-fixation to cereals. Curr. Opin. Biotechnol. 32, 216–222. 10.1016/j.copbio.2015.01.004 25626166

[B13] Glibert P. M. Maranger R. Sobota D. J. Bouwman L. (2014). The Haber Bosch-harmful algal bloom (HB-HAB) link. Environ. Res. Lett. 9:105001 10.1088/1748-9326/9/10/105001

[B14] Good A. G. Beatty P. H. (2011). Fertilizing nature: a tragedy of excess in the commons. PLoS Biol. 9:e1001124. 10.1371/journal.pbio.1001124 21857803 PMC3156687

[B15] Graf M. Schoedl T. Wagner R. (2009). Rationales of gene design and *de novo* gene construction, in Systems Biology and Synthetic Biology, eds Fu P. Panke S. (Hoboken, NJ: John Wiley & Sons, Inc.), 411–438.

[B16] Hondred D. Walker J. M. Mathews D. E. Vierstra R. D. (1999). Use of ubiquitin fusions to augment protein expression in transgenic plants. Plant Physiol. 119, 713–724. 10.1104/pp.119.2.713 9952468 PMC32149

[B17] Hu Y. L. Ribbe M. W. (2013). Nitrogenase assembly. Biochim. Biophys. Acta 1827, 1112–1122. 10.1016/j.bbabio.2012.12.001 23232096 PMC3622157

[B18] Huang S. Taylor N. L. Whelan J. Millar A. H. (2009). Refining the definition of plant mitochondrial presequences through analysis of sorting signals, N-terminal modifications, and cleavage motifs. Plant Physiol. 150, 1272–1285. 10.1104/pp.109.137885 19474214 PMC2705053

[B19] Ivleva N. B. Groat J. Staub J. M. Stephens M. (2016). Expression of active subunit of nitrogenase via integration into plant organelle genome. PLoS ONE 11:e0160951. 10.1371/journal.pone.0160951 27529475 PMC4986947

[B20] Kronzucker H. J. Coskun D. (2015). Bioengineering nitrogen acquisition in rice: promises for global food security, in Biological Nitrogen Fixation, ed de Bruijn F. J. (Hoboken, NJ: John Wiley & Sons, Inc.), 47–56.

[B21] Lahiri S. Pulakat L. Gavini N. (2005). Functional NifD-K fusion protein in *Azotobacter vinelandii* is a homodimeric complex equivalent to the native heterotetrameric MoFe protein. Biochem. Biophys. Res. Commun. 337, 677–684. 10.1016/j.bbrc.2005.09.105 16202390

[B22] Lee S. Lee D. W. Yoo Y. J. Duncan O. Oh Y. J. Lee Y. J.. (2012). Mitochondrial targeting of the Arabidopsis F1-ATPase γ-subunit via multiple compensatory and synergistic presequence motifs. Plant Cell 24, 5037–5057. 10.1105/tpc.112.105361 23250447 PMC3556974

[B23] Lill R. Mühlenhoff U. (2008). Maturation of iron-sulfur proteins in eukaryotes: mechanisms, connected processes, and diseases. Annu. Rev. Biochem. 77, 669–700. 10.1146/annurev.biochem.76.052705.162653 18366324

[B24] López-Torrejón G. Jiménez-Vicente E. Buesa J. M. Hernandez J. A. Verma H. K. Rubio L. M. (2016). Expression of a functional oxygen-labile nitrogenase component in the mitochondrial matrix of aerobically grown yeast. Nat. Commun. 7:11426. 10.1038/ncomms11426 27126134 PMC4855529

[B25] Merrick M. Dixon R. (1984). Why dont plants fix nitrogen. Trends Biotechnol. 2, 162–166. 10.1016/0167-7799(84)90034-9

[B26] Mueller N. D. Gerber J. S. Johnston M. Ray D. K. Ramankutty N. Foley J. A. (2012). Closing yield gaps through nutrient and water management. Nature 490, 254–257. 10.1038/nature11420 22932270

[B27] Murcha M. W. Wang Y. Narsai R. Whelan J. (2014). The plant mitochondrial protein import apparatus - The differences make it interesting. Biochem. Biophys. Acta 1840, 1233–1245. 10.1016/j.bbagen.2013.09.026 24080405

[B28] Naim F. Nakasugi K. Crowhurst R. N. Hilario E. Zwart A. B. Hellens R. P.. (2012). Advanced engineering of lipid metabolism in *Nicotiana benthamiana* using a draft genome and the V2 viral silencing-suppressor protein. PLoS ONE 7:e52717. 10.1371/journal.pone.0052717 23300750 PMC3530501

[B29] Oldroyd G. E. Dixon R. (2014). Biotechnological solutions to the nitrogen problem. Curr. Opin. Biotechnol. 26, 19–24. 10.1016/j.copbio.2013.08.006 24679253

[B30] Ormö M. Cubitt A. B. Kallio K. Gross L. A. Tsien R. Y. Remington S. J. (1996). Crystal structure of the *Aequorea victoria* green fluorescent protein. Science 273, 1392–1395. 10.1126/science.273.5280.1392 8703075

[B31] Petrie J. R. Shrestha P. Belide S. Kennedy Y. Lester G. Liu Q.. (2014). Metabolic engineering *Camelina sativa* with fish oil-like levels of DHA. PLoS ONE 9:e85061. 10.1371/journal.pone.0085061 24465476 PMC3897407

[B32] Poza-Carrion C. Jimenez-Vicente E. Navarro-Rodriguez M. Echavarri-Erasun C. Rubio L. M. (2014). Kinetics of nif gene expression in a nitrogen-fixing bacterium. J. Bacteriol. 196, 595–603. 10.1128/JB.00942-13 24244007 PMC3911164

[B33] Rey L. Maier R. J. (1997). Cytochrome c terminal oxidase pathways of *Azotobacter vinelandii*: analysis of cytochrome c4 and c5 mutants and up-regulation of cytochrome c-dependent pathways with N2 fixation. J. Bacteriol. 179, 7191–7196. 10.1128/jb.179.22.7191-7196.1997 9371471 PMC179665

[B34] Robson R. L. Postgate J. R. (1980). Oxygen and hydrogen in biological nitrogen-fixation. Annu. Rev. Microbiol. 34, 183–207. 10.1146/annurev.mi.34.100180.001151 6776883

[B35] Rockstrom J. Steffen W. Noone K. Persson A. Chapin F. S. Lambin E. F.. (2009). A safe operating space for humanity. Nature 461, 472–475. 10.1038/461472a 19779433

[B36] Rolland V. Badger M. R. Price G. D. (2016). Redirecting the cyanobacterial bicarbonate transporters BicA and SbtA to the chloroplast envelope: soluble and membrane cargos need different chloroplast targeting signals in plants. Front. Plant Sci. 7:185. 10.3389/fpls.2016.00185 26973659 PMC4770052

[B37] Rubio L. M. Ludden P. W. (2008). Biosynthesis of the iron-molybdenum cofactor of nitrogenase. Annu. Rev. Microbiol. 62, 93–111. 10.1146/annurev.micro.62.081307.162737 18429691

[B38] Santi C. Bogusz D. Franche C. (2013). Biological nitrogen fixation in non-legume plants. Ann. Bot. 111, 743–767. 10.1093/aob/mct048 23478942 PMC3631332

[B39] Schmid B. Ribbe M. W. Einsle O. Yoshida M. Thomas L. M. Dean D. R.. (2002). Structure of a cofactor-deficient nitrogenase MoFe protein. Science 296, 352–356. 10.1126/science.1070010 11951047

[B40] Seefeldt L. C. Hoffman B. M. Dean D. R. (2009). Mechanism of Mo-dependent nitrogenase. Annu. Rev. Biochem. 78, 701–722. 10.1146/annurev.biochem.78.070907.103812 19489731 PMC2814439

[B41] Smil V. (2002). Nitrogen and food production: proteins for human diets. Ambio 31, 126–131. 10.1579/0044-7447-31.2.126 12078001

[B42] Studier F. W. Moffatt B. A. (1986). Use of bacteriophage T7 RNA polymerase to direct selective high-level expression of cloned genes. J. Mol. Biol. 189, 113–130. 10.1016/0022-2836(86)90385-2 3537305

[B43] Suh M.-H. Pulakat L. Gavini N. (2003). Functional expression of a fusion-dimeric mofe protein of nitrogenase in *Azotobacter vinelandii*. J. Biol. Chem. 278, 5353–5360. 10.1074/jbc.M208969200 12468552

[B44] Sutton M. A. Erisman J. W. Dentener F. Moller D. (2008). Ammonia in the environment: from ancient times to the present. Environ. Pollut. 156, 583–604. 10.1016/j.envpol.2008.03.013 18499318

[B45] Temme K. Zhao D. Voigt C. A. (2012). Refactoring the nitrogen fixation gene cluster from Klebsiella oxytoca. Proc. Natl. Acad. Sci. U.S.A. 109, 7085–7090. 10.1073/pnas.1120788109 22509035 PMC3345007

[B46] Wood C. C. Petrie J. R. Shrestha P. Mansour M. P. Nichols P. D. Green A. G.. (2009). A leaf-based assay using interchangeable design principles to rapidly assemble multistep recombinant pathways. Plant Biotechnol. J. 7, 914–924. 10.1111/j.1467-7652.2009.00453.x 19843252

[B47] Yang J. Liu X. Yang X. Zhang M. (2010). Mitochondrially-targeted expression of a cytoplasmic male sterility-associated orf220 gene causes male sterility in *Brassica juncea*. BMC Plant Biol. 10:231. 10.1186/1471-2229-10-231 20974011 PMC3017852

[B48] Yuvaniyama P. Agar J. N. Cash V. L. Johnson M. K. Dean D. R. (2000). NifS-directed assembly of a transient [2Fe-2S] cluster within the NifU protein. Proc. Natl. Acad. Sci. U.S.A. 97, 599–604. 10.1073/pnas.97.2.599 10639125 PMC15376

