# Supplementary material for: Expression of 16 Nitrogenase Proteins within the Plant Mitochondrial Matrix
Source: Front Plant Sci. 2017 Mar 3;8:287. doi: 10.3389/fpls.2017.00287 (PMC5334340; doi:10.3389/fpls.2017.00287)
Supplement: Supplementary file 1 [file Table1.docx]

**Supplementary Table 1**

**List of GFP/Nif constructs used in this study including protein sequence descriptions**

| **Nif polypeptide** | **Acc #** | **Construct ID** | **Encoded polypeptide** | **Codon optimization** | **Epitope** | **Mol Wt (unprocessed) (kDa)** | **Mol Wt (processed) (kDa)** |
| --- | --- | --- | --- | --- | --- | --- | --- |
| **none** | ABG78037 | pRA01 | pFAγ::GFP | N/A | none | 35.7 | 31 |
| **none** | ABG78037 | pRA21 | mFAγ::GFP | N/A | none | 35.7 | 35.7 |
| **B** | P10390 | pRA03 | pFAγ::NifB::HA | human | HA | 61 | 56.3 |
| **D** | P00466 | pRA07 | pFAγ::NifD::FLAG | human | FLAG | 63.8 | 59.1 |
| **E** | P08737 | pRA09 | pFAγ::NifE::HA | human | HA | 60.3 | 55.6 |
| **F** | P04668 | pRA05 | pFAγ::NifF::HA | Arabidopsis | HA | 29 | 24.3 |
| **H** | P00458 | pRA10 | pFAγ::NifH::HA | human | HA | 42 | 37.3 |
| **J** | P03833 | pRA06 | pFAγ::NifJ::FLAG | Arabidopsis | FLAG | 137.9 | 133.2 |
| **K** | P09772 | pRA11 | pFAγ::NifK::HA | human | HA | 68 | 63.3 |
| **M** | P0A3Y9 | pRA18 | pFAγ::NifM::HA | Arabidopsis | HA | 40.6 | 35.9 |
| **N** | P08738 | pRA13 | pFAγ::NifN::FLAG | human | FLAG | 60.3 | 55.6 |
| **Q** | P10392 | pRA08 | pFAγ::NifQ::HA | human | HA | 29.8 | 25.1 |
| **S** | CAA31675 | pRA16 | pFAγ::NifS::HA | human | HA | 53.3 | 48.6 |
| **U** | P05343 | pRA15 | pFAγ::NifU::FLAG | human | FLAG | 39.4 | 34.7 |
| **V** | P05345 | pRA17 | pFAγ::NifV::FLAG | Arabidopsis | FLAG | 51 | 46.3 |
| **X** | P09136 | pRA14 | pFAγ::NifX::FLAG | human | FLAG | 28 | 23.3 |
| **Y** | CAA31670 | pRA12 | pFAγ::NifY::HA | human | HA | 34.9 | 30.2 |
| **Z** | P0A3U2 | pRA04 | pFAγ::NifZ::FLAG | Arabidopsis | FLAG | 26.5 | 21.8 |
| **D** | P00466 | pRA19 | pFAγ::NifD::HA | human | HA | 63.8 | 59.1 |
| **D** | P00466 | pRA22 | mFAγ::NifD::HA | human | HA | 63.8 | 63.8 |
| **D** | P00466 | pRA24 | pFAγ::NifD::HA | Arabidopsis | HA | 63.8 | 59.1 |
| **DK** | P00466/ P09772 | pRA20 | pFAγ::NifD-linker-K::HA | Arabidopsis | FLAG + HA | 124 | 119.3 |

**Descriptions of protein coding regions for constructs used in this study:**

pFAγ::GFP in **pRA1**

Derived from GFP S65T

**N terminal modifications:** 77 a.a pFAγ (red sequence)as described in Lee et al 2009 fused in frame, followed by “GAP” (blue) for cloning purposes.

mamavfrregrrllpsiaarpiaairsplssdqeegllgvrsistqvvrnrmksvkniqkitkamkmvaasklravqgapmvskgeelftgvvpilveldgdvnghkfsvsgegegdatygkltlkficttgklpvpwptlvttftygvqcfsrypdhmkqhdffksampegyvqertiffkddgnyktraevkfegdtlvnrielkgidfkedgnilghkleynynshnvyimadkqkngikvnfkirhniedgsvqladhyqqntpigdgpvllpdnhylstqsalskdpnekrdhmvllefvtaagitlgmdelyk

mFAγ::GFP in **pRA21**

Derived from GFP S65T

**N terminal modifications:** 77 a.a mFAγ (red sequence) fused in frame, followed by “GAP” (blue)for cloning purposes.

mamavfrregraaaaaaaarpiaaaaaaaaaaaeegllaaaaaaaavvrnrmksvkniqkitkamkmvaasklravqgapmvskgeelftgvvpilveldgdvnghkfsvsgegegdatygkltlkficttgklpvpwptlvttftygvqcfsrypdhmkqhdffksampegyvqertiffkddgnyktraevkfegdtlvnrielkgidfkedgnilghkleynynshnvyimadkqkngikvnfkirhniedgsvqladhyqqntpigdgpvllpdnhylstqsalskdpnekrdhmvllefvtaagitlgmdelyk

pFAγ::NifB::HA **in pRA3**

Derived from: *Klebsiella pneumoniae* NifB

**N terminal modifications:** First Met removed and replaced with 77 a.a pFAγ (red sequence) as described in Lee et al 2009, followed by “GAP” (blue) for cloning purposes.

**C terminal modifications:** additional residues included (blue) and HA tag (green)

mamavfrregrrllpsiaarpiaairsplssdqeegllgvrsistqvvrnrmksvkniqkitkamkmvaasklravqgaptscssfsggkacrpaddsaltplvadkaaahpcysrhghhrfarmhlpvapacnlqcnycnrkfdcsnesrpgvsstlltpeqavvkvrqvaqaipqlsvvgiagpgdplaniartfrtlelireqlpdlklclstnglvlpdavdrlldvgvdhvtvtintldaeiaaqiyawlwldgerysgreageiliarqlegvrrltakgvlvkinsvlipgindsgmagvsralrasgafihnimpliarpehgtvfglngqpepdaetlaatrsrcgevmpqmthchqcradaigmlgedrsqqftqlpapeslpawlpilhqraqlhasiatrgeseaddaclvavassrgdvidchfghadrfyiyslsaagmvlvnerftpkycqgrddcepqdnaarfaailelladvkavfcvrightpwqqleqegiepcvdgawrpvsevlpawwqqrrgswpaalphkgvaagggggypydvpdyapg

pFAγ::NifD::FLAG **in pRA7**

Derived from *Klebsiella pneumoniae* NifD

**N terminal modifications:** First two Mets removed and replaced with 77 a.a pFAγ (red sequence)as described in Lee et al 2009, followed by “GAP” (Blue) for cloning purposes.

**C terminal modifications:** additional residues included (Blue) and FLAG tag (green)

mamavfrregrrllpsiaarpiaairsplssdqeegllgvrsistqvvrnrmksvkniqkitkamkmvaasklravqgaptnatgernlaliqevlevfpetarkerrkhmmvsdpkmksvgkciisnrksqpgvmtvrgcayagskgvvfgpikdmahishgpagcgqysraerrnyytgvsgvdsfgtlnftsdfqerdivfggdkklsklieemellfpltkgitiqsecpvgligddisavanasskaldkpvipvrcegfrgvsqslghhiandvvrdwilnnregqpfettpydvaiigdyniggdawasrilleemglrvvaqwsgdgtlvementpfvklnlvhcyrsmnyiarhmeekhqipwmeynffgptkiaeslrkiadqfddtiranaeaviaryegqmaaiiakyrprlegrkvllyigglrprhvigayedlgmeiiaagyefahnddydrtlpdlkegtllfddassyeleafvkalkpdligsgikekyifqkmgvpfrqmhswdysgpyhgydgfaifardmdmtlnnpawneltapwlksaagdykddddkpg

pFAγ::NifE::HA **in pRA9**

Derived from: *Klebsiella pneumoniae* NifE

**N terminal modifications:** First Met removed and replaced with 77 a.a pFAγ (red sequence)as described in Lee et al 2009, followed by “GAP” (Blue) for cloning purposes.

**C terminal modifications:** additional residues included (Blue) and HA tag (green)

mamavfrregrrllpsiaarpiaairsplssdqeegllgvrsistqvvrnrmksvkniqkitkamkmvaasklravqgapkgneilalldepacehnhkqksgcsapkpgataagcafdgaqitllpiadvahlvhgpigcagsswdnrgsassgptlnrlgfttdlneqdvimgrgerrlfhavrhivtryhpaavfiyntcvpamegddleavcqaaqtatgvpviaidaagfygsknlgnrpagdvmvkrvigqrepapwpestlfapeqrhdigligefniagefwhiqplldelgirvlgslsgdgrfaeiqtmhraqanmlvcsralinvaraleqrygtpwfegsfygiratsdalrqlaallgdddlrqrtealiareeqaaelalqpwreqlrgrkallytggvkswsvvsalqdlgmtvvatgtrksteedkqrirelmgeeavmleegnartlldvvyryqadlmiaggrnmytaykarlpfldinqerehafagyqgivtlarqlcqtinspiwpqthsrapwragggggypydvpdyapg

pFAγ::NifF::HA **in pRA5**

Derived from: *Klebsiella pneumoniae* NifF

**N terminal modifications:** 77 a.a pFAγ (red sequence)as described in Lee et al 2009 fused in frame, followed by “GAP” (Blue) for cloning purposes.

**C terminal modifications:** additional residues included (Blue) and HA tag (green)

mamavfrregrrllpsiaarpiaairsplssdqeegllgvrsistqvvrnrmksvkniqkitkamkmvaasklravqgapmanigiffgtdtgktrkiakmihkqlgeladapvninrttlddfmaypvlllgtptlgdgqlpgleagceseswsefisglddaslkgktvalfglgdqrgypdnfvsgmrplfdalsargaqmigswpnegyefsassalegdrfvglvldqdnqfdqtearlaswleeikrtvlypydvpdyapg

pFAγ::NifH::HA **in pRA10**

Derived from: *Klebsiella pneumoniae* NifH

N terminal modifications: First Met removed and replaced with 77 a.a pFAγ (red sequence)as described in Lee et al 2009, followed by “GAP” (Blue) for cloning purposes.

C terminal modifications: additional residues included (Blue) and HA tag (green)

mamavfrregrrllpsiaarpiaairsplssdqeegllgvrsistqvvrnrmksvkniqkitkamkmvaasklravqgaptmrqcaiygkggigkstttqnlvaalaemgkkvmivgcdpkadstrlilhakaqntimemaaevgsvedleledvlqigygdvrcaesggpepgvgcagrgvitainfleeegayeddldfvfydvlgdvvcggfampirenkaqeiyivcsgemmamyaanniskgivkyaksgkvrlgglicnsrqtdredeliialaeklgtqmihfvprdnivqraeirrmtvieydpackqaneyrtlaqkivnntmkvvptpctmdelesllmefgimeeedtsiigktaaeenaaagggggypydvpdyapg

pFAγ::NifJ::FLAG **in pRA6 (RA synth)**

Derived from: *Klebsiella pneumoniae* NifK

**Notes: N terminal modifications:** 77 a.a pFAγ (red sequence)as described in Lee et al 2009 fused in frame, followed by “GAP” (Blue) for cloning purposes.

**C terminal modifications:** additional residues included (Blue) and FLAG tag (green)

mamavfrregrrllpsiaarpiaairsplssdqeegllgvrsistqvvrnrmksvkniqkitkamkmvaasklravqgapmsgkmktmdgnaaaawisyaftevaaiypitpstpmaenvdewaaqgkknlfgqpvrlmemqseagaagavhgalqagaltttytasqglllmipnmykiagellpgvfhvsaralatnslnifgdhqdvmavrqtgcamlaennvqqvmdlsavahlaaikgripfvnffdgfrtsheiqkievleyeqlatlldrpaldsfrrnalhpdhpvirgtaqnpdiyfqereagnrfyqalpdivesymtqisaltgreyhlfnytgaadaerviiamgsvcdtvqevvdtlnaagekvgllsvhlfrpfslahffaqlpktvqriavldrtkepgaqaeplcldvknafyhhddaplivggryalggkdvlpndiaavfdnlnkplpmdgftlgivddvtftslpprqqtlavshdgitackfwgmgsdgtvganksaikiigdktplyaqayfsydskksggitvshlrfgdrpinspylihradfiscsqqsyverydlldglkpggtfllncswsdaeleqhlpvgfkrylarenihfytlnavdiarelglggrfnmlmqaaffklaaiidpqtaadylkqaveksygskgaaviemnqraielgmaslhqvtipahwatldepaaqasammpdfirdilqpmnrqcgdqlpvsafvgmedgtfpsgtaawekrgialevpvwqpegctqcnqcaficphaairpallngeehdaapvgllskpaqgakeyhyhlaispldcsgcgncvdicpargkalkmqsldsqrqmapvwdyalaltpksnpfrkttvkgsqfetpllefsgacagcgetpyarlitqlfgdrmlianatgcssiwgasapsipyttnhrghgpawanslfednaefglgmmlggqavrqqiaddmtaalalpvsdelsdamrqwlakqdegegtreradrlserlaaekegvplleqlwqnrdyfvrrsqwifggdgwaydigfggldhvlasgedvnilvfdtevysntggqsskstpvaaiakfaaqgkrtrkkdlgmmamsygnvyvaqvamgadkdqtlraiaeaeawpgpslviayaacinhglkagmrcsqreakraveagywhlwryhpqreaegktpfmldseepeesfrdfllgevryaslhkttphladalfsrteedararfaqyrrlageedykddddkpg

pFAγ::NifK::HA **in pRA11**

Derived from: *Klebsiella pneumoniae* NifK

**N terminal modifications:** First Met removed and replaced with 77 a.a pFAγ (red sequence)as described in Lee et al 2009, followed by “GAP” (Blue) for cloning purposes.

**C terminal modifications:** additional residues included (Blue) and HA tag (green)

mamavfrregrrllpsiaarpiaairsplssdqeegllgvrsistqvvrnrmksvkniqkitkamkmvaasklravqgapsqtidkinscyplfeqdeyqelfrnkrqleeahdaqrvqevfawtttaeyealnfrrealtvdpakacqplgavlcslgfantlpyvhgsqgcvayfrtyfnrhfkepiacvsdsmtedaavfggnnnmnlglqnasalykpeiiavsttcmaevigddlqafianakkdgfvdssiavphahtpsfigshvtgwdnmfegfaktftadyqgqpgklpklnlvtgfetylgnfrvlkrmmeqmavpcsllsdpsevldtpadghyrmysggttqqemkeapdaidtlllqpwqllkskkvvqemwnqpatevaiplglaatdellmtvsqlsgkpiadaltlergrlvdmmldshtwlhgkkfglygdpdfvmgltrfllelgceptvilshnankrwqkamnkmldaspygrdsevfincdlwhfrslmftrqpdfmignsygkfiqrdtlakgkafevplirlgfplfdrhhlhrqttwgyegamnivttlvnavlekldsdtsqlgktdysfdlvragggggypydvpdyapg

pFAγ::NifM::HA **in pRA18**

Derived from: *Klebsiella pneumoniae* NifM

**N terminal modifications:** 77 a.a pFAγ (red sequence)as described in Lee et al 2009 fused in frame, followed by “GAP” (Blue)for cloning purposes.

**C terminal modifications:** additional residues included (Blue) and HA tag (green)

mamavfrregrrllpsiaarpiaairsplssdqeegllgvrsistqvvrnrmksvkniqkitkamkmvaasklravqgapmnpwqrfarqrlarsrwnrdpaaldpadtpafeqawqrqchmeqtivarvpegdipaalleniaaslaiwldegdfapperaaivrhharlelafadiarqapqpdlstvqawylrhqtqfmrpeqrltrhllltvdndreavhqrilglyrqinasrdafaplaqrhshcpsaleegrlgwisrgllypqletalfslaenalslpiaselgwhllwceairpaapmepqqalesardylwqqsqqrhqrqwleqmisrqpglcgypydvpdyapg

pFAγ::NifN::FLAG **in pRA13**

Derived from: *Klebsiella pneumoniae* NifN

**N terminal modifications:** First Met removed and replaced with 77 a.a pFAγ (red sequence)as described in Lee et al 2009, followed by “GAP” (Blue) for cloning purposes.

**C terminal modifications**: native terminal Aspartic Acid residue (D) has been removed. Additional residues included (Blue) and FLAG tag (green)

mamavfrregrrllpsiaarpiaairsplssdqeegllgvrsistqvvrnrmksvkniqkitkamkmvaasklravqgapadifrtdkplavspiktgqplgailaslgiehsiplvhgaqgcsafakvffiqhfhdpvplqstamdptstimgadgniftaldtlcqrnnpqaivllstglseaqgsdisrvvrqfreeyprhkgvailtvntpdfygsmengfsavlesvieqwvppaprpaqrnrrvnllvshlcspgdiewlrrcveafglqpiilpdlaqsmdghlaqgdfspltqggtplrqieqmgqslcsfaigvslhrassllaprcrgevialphlmtlercdafihqlakisgravpewlerqrgqlqdamidchmwlqgqrmaiaaegdllaawcdfansqgmqpgplvaptghpslrqlpvervvpgdledlqtllcahpadllvanshardlaeqfalplvragfplfdklgefrrvrqgysgmrdtlfelanlirerhhhlahyrsplrqnpesslstggayaaagdykddddkpg

pFAγ::NifQ::HA **in pRA8**

Derived from: *Klebsiella pneumoniae* NifQ

N terminal modifications: First Met removed and replaced with 77 a.a pFAγ (red sequence)as described in Lee et al 2009, followed by “GAP” (Blue) for cloning purposes.

**C terminal modifications:** additional residues included (Blue) and HA tag (green)

mamavfrregrrllpsiaarpiaairsplssdqeegllgvrsistqvvrnrmksvkniqkitkamkmvaasklravqgapppldwlrrlwllyhagkgsfplrmglsprdwqalrrrlgevetpldgetltrrrlmaelnatreeerqqlgawlagwmqqdagpmaqiiaevslafnhlwqdlglasraelrllmsdcfpqlvvmnehnmrwkkffyrqrcllqqgevicrspscdecwersacfeagggggypydvpdyapg

pFAγ::NifS::HA **in pRA16**

Derived from: *Klebsiella pneumoniae* NifS

N terminal modifications: First Met removed and replaced with 77 a.a pFAγ (red sequence)as described in Lee et al 2009, followed by “GAP” (Blue) for cloning purposes.

**C terminal modifications:** additional residues included (Blue) and HA tag (green)

mamavfrregrrllpsiaarpiaairsplssdqeegllgvrsistqvvrnrmksvkniqkitkamkmvaasklravqgapkqvyldnnattrldpmvleammpfltdfygnpssihdfgipaqaalerahqqaaallgaeypseiiftscateatataiasaiallperreiitsvvehpatlaacehmeregyrihriavdgegaldmaqfraalsprvalvsvmwannetgvlfpigemaelaheqgalfhcdavqvvgkipiavgqtridmlscsahkfhgpkgvgclylrrgtrfrpllrgghqeygrragtenicgivgmgaacelanihlpgmthigqlrnrlehrllasvpsvmvmgggqpavpgtvnlafefiegeaillllnqagiaassgsactsgslepshvmramnipytaahgtirfslsrytrekeidyvvatlppiidrlralspywqngkprpadavftpvygagggggypydvpdyapg

pFAγ::NifU::FLAG **in pRA15**

Derived from: *Klebsiella pneumoniae* NifU

**N terminal modifications:** First Met removed and replaced with 77 a.a pFAγ (red sequence)as described in Lee et al 2009, followed by “GAP” (Blue) for cloning purposes.

**C terminal modifications:** additional residues included (Blue) and HA tag (green)

mamavfrregrrllpsiaarpiaairsplssdqeegllgvrsistqvvrnrmksvkniqkitkamkmvaasklravqgapwnysekvkdhffnprnarvvdnanavgdvgslscgdalrlmlrvdpqseiieeagfqtfgcgsaiasssalteliightlaeagqitnqqiadyldglppekmhcsvmgqealraaianfrgesleeehdegklickcfgvdeghirravqnnglttlaevinytkagggctschekielalaeilaqqpqttpavasgkdphwqsvvdtiaelrphiqadggdmallsvtnhqvtvslsgscsgcmmtdmtlawlqqklmertgcymevvaaagdykddddkpg

pFAγ::NifV::FLAG **in pRA17**

Derived from: *Klebsiella pneumoniae* NifV

**N terminal modifications:** 77 a.a pFAγ (red sequence)as described in Lee et al 2009 fused in frame, followed by “GAP” (Blue) for cloning purposes.

**C terminal modifications:** additional residues included (Blue) and FLAG tag (green)

mamavfrregrrllpsiaarpiaairsplssdqeegllgvrsistqvvrnrmksvkniqkitkamkmvaasklravqgapmervlindttlrdgeqspgvafrtsekvaiaealyaagitamevgtpamgdeeiariqlvrrqlpdatlmtwcrmnaleirqsadlgidwvdisipasdklrqyklreplavllerlamfihlahtlglkvcigcedasrasgqtlraiaevaqnapaarlryadtvglldpfttaaqisalrdvwsgeiemhahndlgmatantlaavsagatsvnttvlglgeragnaaawkpsalglerclgvetgvhfsalpalcqrvaeaaqraidpqqplvgelvfthesgvhvaallrdsesyqsiapslmgrsyrlvlgkhsgrqavngvfdqmgyhlnaaqinqllpairrfaenwkrspkdyelvaiydelcgesalrargdykddddkpg

pFAγ::NifX::FLAG **in pRA14**

Derived from: *Klebsiella pneumoniae* NifX

**N terminal modifications:** First Met removed and replaced with 77 a.a pFAγ (red sequence)as described in Lee et al 2009, followed by “GAP” (Blue) for cloning purposes.

**C terminal modifications:** additional residues included (Blue) and HA tag (green)

mamavfrregrrllpsiaarpiaairsplssdqeegllgvrsistqvvrnrmksvkniqkitkamkmvaasklravqgapppinrqfdmvhsdewsmkvafassdyrhvdqhfgatprlvvygvkadrvtlirvvdfsvenghqtekiarrihaledcvtlfcvaigdavfrqllqvgvraervpadttivgllqeiqlywydkgqrknqrqrdperftrllqeqewhgdpdprragdykddddkpg

pFAγ::NifY::HA **in pRA12**

Derived from: *Klebsiella pneumoniae* NifY

N terminal modifications: First Met removed and replaced with 77 a.a pFAγ (red sequence) as described in Lee et al 2009, followed by “GAP” (Blue) for cloning purposes.

**C terminal modifications:** additional residues included (Blue) and HA tag (green)

mamavfrregrrllpsiaarpiaairsplssdqeegllgvrsistqvvrnrmksvkniqkitkamkmvaasklravqgapsdndtlfwrmlalfqslpdlqpaqivdwlaqesgetltperlatltqpqlaasfpsatavmsparwsrvmaslqgalpahlrivrpaqrtpqllaafcsqdglvinghfgqgrlffiyafdeqggwlydlrrypsaphqqeanevrarliedcqllfcqeiggpaaarpirhrihpmkaqpgttiqaqceaintllagrlppwlakrlnrdnpleervfagggggypydvpdyapg

pFAγ::NifZ::FLAG **in pRA4**

Derived from: *Klebsiella pneumoniae* NifM

**N terminal modifications:** 77 a.a pFAγ (red sequence)as described in Lee et al 2009 fused in frame, followed by “GAP” (Blue) for cloning purposes.

**C terminal modifications:** additional residues included (Blue) and FLAG tag (green)

mamavfrregrrllpsiaarpiaairsplssdqeegllgvrsistqvvrnrmksvkniqkitkamkmvaasklravqgapmrpkftfseevrvvrairndgtvagfapgallvrrgstgfvrdwgvflqdqiiyqihfpetdriigcreqelipitqpwlagnlqyrdsvtcqmalavngdvvvsagqrgrveatdrgelgdsytvdfsgrwfrvpvqaialieereedykddddkpg

**Nif D variants:**

pFAγ::NifD::HA **in pRA19**

Derived from *Klebsiella pneumoniae* NifD

**N terminal modifications:** First two Mets removed and replaced with 77 a.a pFAγ (red sequence)as described in Lee et al 2009, followed by “GAP” (Blue) for cloning purposes.

**C terminal modifications:** additional residues included (Blue) and HA tag (green)

**Other:** identical to pRA7 except HA tag in place of FLAG tag

mamavfrregrrllpsiaarpiaairsplssdqeegllgvrsistqvvrnrmksvkniqkitkamkmvaasklravqgaptnatgernlaliqevlevfpetarkerrkhmmvsdpkmksvgkciisnrksqpgvmtvrgcayagskgvvfgpikdmahishgpagcgqysraerrnyytgvsgvdsfgtlnftsdfqerdivfggdkklsklieemellfpltkgitiqsecpvgligddisavanasskaldkpvipvrcegfrgvsqslghhiandvvrdwilnnregqpfettpydvaiigdyniggdawasrilleemglrvvaqwsgdgtlvementpfvklnlvhcyrsmnyiarhmeekhqipwmeynffgptkiaeslrkiadqfddtiranaeaviaryegqmaaiiakyrprlegrkvllyigglrprhvigayedlgmeiiaagyefahnddydrtlpdlkegtllfddassyeleafvkalkpdligsgikekyifqkmgvpfrqmhswdysgpyhgydgfaifardmdmtlnnpawneltapwlksaagypydvpdyapg

mFAγ::NifD::HA **in pRA22**

Derived from *Klebsiella pneumoniae* NifD

**N terminal modifications:** First two Mets removed and replaced with a extensively modified version of the 77 a.a pFAγ (red sequence)as described in Lee et al 2009, followed by “GAP” (Blue) for cloning purposes.

**C terminal modifications:** additional residues included (Blue) and HA tag (green)

**Other:** identical to pRA19 except for modified pFAγ to prevent matrix processing

mamavfrregraaaaaaaarpiaaaaaaaaaaaeegllaaaaaaaavvrnrmksvkniqkitkamkmvaasklravqgaptnatgernlaliqevlevfpetarkerrkhmmvsdpkmksvgkciisnrksqpgvmtvrgcayagskgvvfgpikdmahishgpagcgqysraerrnyytgvsgvdsfgtlnftsdfqerdivfggdkklsklieemellfpltkgitiqsecpvgligddisavanasskaldkpvipvrcegfrgvsqslghhiandvvrdwilnnregqpfettpydvaiigdyniggdawasrilleemglrvvaqwsgdgtlvementpfvklnlvhcyrsmnyiarhmeekhqipwmeynffgptkiaeslrkiadqfddtiranaeaviaryegqmaaiiakyrprlegrkvllyigglrprhvigayedlgmeiiaagyefahnddydrtlpdlkegtllfddassyeleafvkalkpdligsgikekyifqkmgvpfrqmhswdysgpyhgydgfaifardmdmtlnnpawneltapwlksaagypydvpdyapg

pFAγ::NifD::HA **in pRA24**

Derived from *Klebsiella pneumoniae* NifD

**N terminal modifications:** First two Mets removed and replaced with 77 a.a pFAγ (red sequence)as described in Lee et al 2009, followed by “GAP” (Blue) for cloning purposes.

**C terminal modifications:** additional residues included (Blue) and HA tag (green)

**Other:** Identical to pRA7 except HA tag in place of FLAG tag. Protein sequence identical to pRA19, but different (Arabidopsis) codon optimisation for NifD coding sequence

mamavfrregrrllpsiaarpiaairsplssdqeegllgvrsistqvvrnrmksvkniqkitkamkmvaasklravqgaptnatgernlaliqevlevfpetarkerrkhmmvsdpkmksvgkciisnrksqpgvmtvrgcayagskgvvfgpikdmahishgpagcgqysraerrnyytgvsgvdsfgtlnftsdfqerdivfggdkklsklieemellfpltkgitiqsecpvgligddisavanasskaldkpvipvrcegfrgvsqslghhiandvvrdwilnnregqpfettpydvaiigdyniggdawasrilleemglrvvaqwsgdgtlvementpfvklnlvhcyrsmnyiarhmeekhqipwmeynffgptkiaeslrkiadqfddtiranaeaviaryegqmaaiiakyrprlegrkvllyigglrprhvigayedlgmeiiaagyefahnddydrtlpdlkegtllfddassyeleafvkalkpdligsgikekyifqkmgvpfrqmhswdysgpyhgydgfaifardmdmtlnnpawneltapwlksaagypydvpdyapg

pFAγ::NifD-linker-K::HA **in pRA20**

Derived from *Klebsiella pneumoniae* NifD and NifK

**N terminal modifications:** First two Mets of NifD (purple seq) removed and replaced with 77 a.a pFAγ (red sequence)as described in Lee et al 2009, followed by “GAP” for cloning purposes.

**C terminal modifications:** HA tag appended at native C terminus of NifK (green).

**Other:** Linker sequence (orange) from 11-residue section of unstructured linker region from *hypocrea jecorina* cellobiohydrolase II (Accession no. AAG39980.1, ATPPPGSTTTR) with the final arginine replaced by an alanine, then an 8-residue FLAG-tag (black)followed by another copy of the 11-residue unstructured linker sequence with the arginine replaced by an alanine.

First Met removed from NifK sequence (pink seq).

mamavfrregrrllpsiaarpiaairsplssdqeegllgvrsistqvvrnrmksvkniqkitkamkmvaasklravqgaptnatgernlaliqevlevfpetarkerrkhmmvsdpkmksvgkciisnrksqpgvmtvrgcayagskgvvfgpikdmahishgpagcgqysraerrnyytgvsgvdsfgtlnftsdfqerdivfggdkklsklieemellfpltkgitiqsecpvgligddisavanasskaldkpvipvrcegfrgvsqslghhiandvvrdwilnnregqpfettpydvaiigdyniggdawasrilleemglrvvaqwsgdgtlvementpfvklnlvhcyrsmnyiarhmeekhqipwmeynffgptkiaeslrkiadqfddtiranaeaviaryegqmaaiiakyrprlegrkvllyigglrprhvigayedlgmeiiaagyefahnddydrtlpdlkegtllfddassyeleafvkalkpdligsgikekyifqkmgvpfrqmhswdysgpyhgydgfaifardmdmtlnnpawneltapwlksaatpppgstttadykddddkatpppgstttasqtidkinscyplfeqdeyqelfrnkrqleeahdaqrvqevfawtttaeyealnfrrealtvdpakacqplgavlcslgfantlpyvhgsqgcvayfrtyfnrhfkepiacvsdsmtedaavfggnnnmnlglqnasalykpeiiavsttcmaevigddlqafianakkdgfvdssiavphahtpsfigshvtgwdnmfegfaktftadyqgqpgklpklnlvtgfetylgnfrvlkrmmeqmavpcsllsdpsevldtpadghyrmysggttqqemkeapdaidtlllqpwqllkskkvvqemwnqpatevaiplglaatdellmtvsqlsgkpiadaltlergrlvdmmldshtwlhgkkfglygdpdfvmgltrfllelgceptvilshnankrwqkamnkmldaspygrdsevfincdlwhfrslmftrqpdfmignsygkfiqrdtlakgkafevplirlgfplfdrhhlhrqttwgyegamnivttlvnavlekldsdtsqlgktdysfdlvrYPYDVPDYA

**Table S2 Primers used in this study**

| **primer NAME** | **SEqeunce 5’ -> 3’** |
| --- | --- |
| NB_GADPH_F | cactaccaactgccttgcac |
| NB_GADPH_R | atgaagcagctcttccacct |

| MTP_F | ATGGCAATGGCTGTTTTCCGTCGCG |
| --- | --- |
| MTP_R | CTTAACACTCTTCATGCGGTTACGCACCAC |
