# Supplementary figures and images for: Expression of 16 Nitrogenase Proteins within the Plant Mitochondrial Matrix
Source: Front Plant Sci. 2017 Mar 3;8:287. doi: 10.3389/fpls.2017.00287 (PMC5334340; doi:10.3389/fpls.2017.00287)

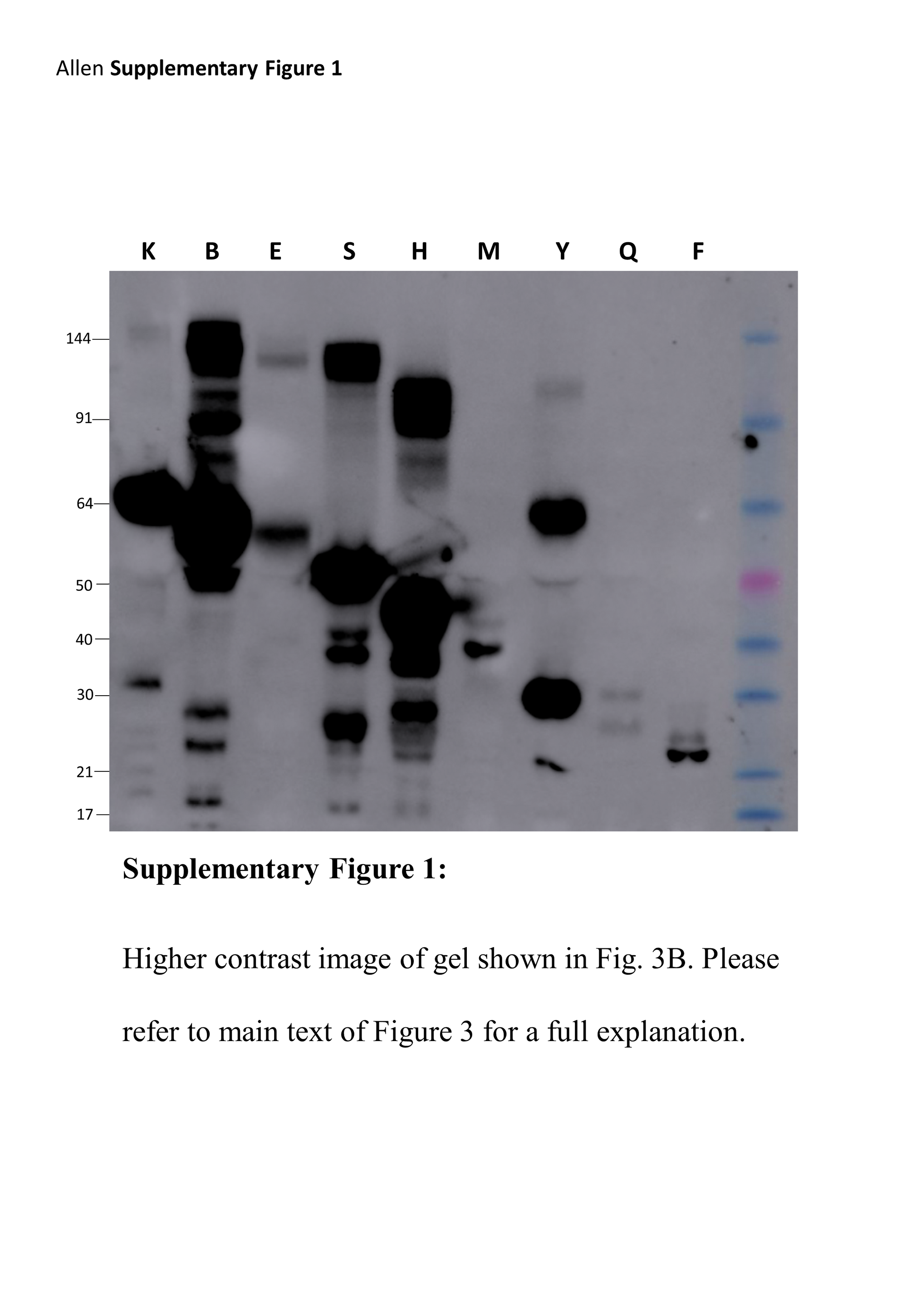

Supplement: Supplementary file 2 [file Image1.TIF]

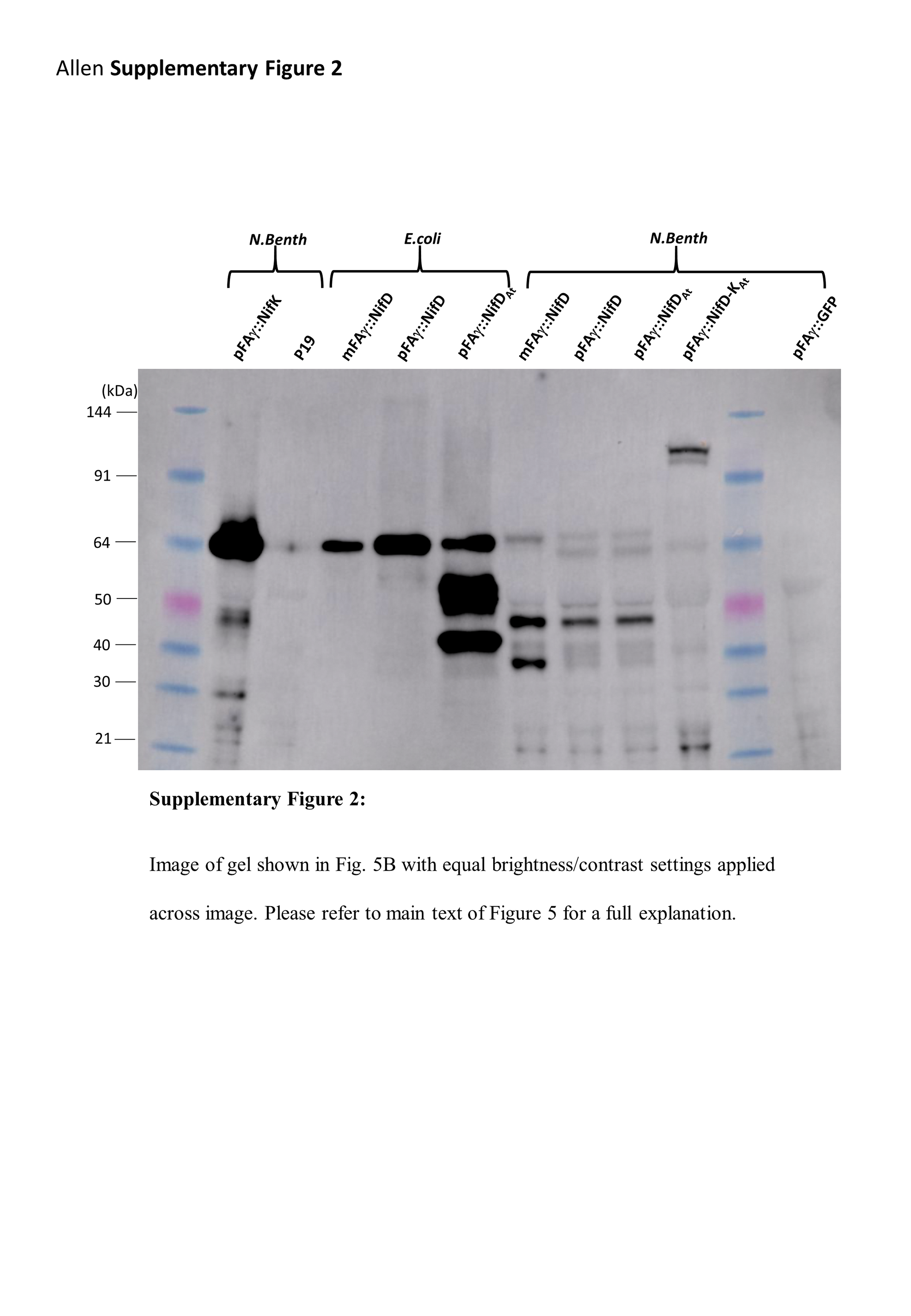

Supplement: Supplementary file 3 [file Image2.TIF]
